# Actor–critic networks with analogue memristors mimicking reward-based learning

**DOI:** 10.1038/s42256-025-01149-w

**Published:** 2025-12-09

**Authors:** Kevin Portner, Till Zellweger, Flavio Martinelli, Laura Bégon-Lours, Valeria Bragaglia, Christoph Weilenmann, Daniel Jubin, Donato Francesco Falcone, Felix Hermann, Oscar Hrynkevych, Tommaso Stecconi, Antonio La Porta, Ute Drechsler, Antonis Olziersky, Bert Jan Offrein, Wulfram Gerstner, Mathieu Luisier, Alexandros Emboras

**Affiliations:** 1 Integrated Systems Laboratory, ETH Zurich, Zurich, Switzerland; 2 School of Life Sciences and School of Computer and Communication Sciences, École Polytechnique Fédérale de Lausanne, Lausanne EPFL, Switzerland; 3 IBM Research Europe—Zurich, Rüschlikon, Switzerland

**Keywords:** Electronic devices, Computational science, Network models

## Abstract

Advancements in memristive devices have given rise to a new generation of specialized hardware for bio-inspired computing. However, most of these implementations draw only partial inspiration from the architecture and functionalities of the mammalian brain. Moreover, the use of memristive hardware is typically restricted to specific elements within the learning algorithm, leaving computationally expensive operations to be executed in software. Here we demonstrate reinforcement learning through an actor–critic temporal difference algorithm implemented on analogue memristors, mirroring the principles of reward-based learning in a neural network architecture similar to the one found in biology. Memristors are used as multipurpose elements within the learning algorithm: they act as synaptic weights that are trained online, they calculate the weight updates associated with the temporal difference error directly in hardware and they determine the actions to navigate the environment. Owing to these features, weight training can take place entirely in memory, eliminating data movement. We test our framework on two navigation tasks—the T-maze and the Morris water maze—using analogue memristors based on the valence change memory effect. Our approach represents the first step towards fully in-memory and online neuromorphic computing engines based on bio-inspired learning schemes.

## Abstract (web summary)

A framework based on actor–critic temporal difference learning and employing a biologically plausible network architecture that mimics reward-based learning on memristors and enables full in-memory training for navigation tasks is discussed.

## Main

With its ability to adapt to new situations, process large amounts of data and generalize from past experiences, the human brain is a beacon of efficiency and computational power. Adjustments in brain connectivity are governed by ‘learning rules’^1^ that can be classified as two- or three-factor rules^2^. Although two-factor rules such as Hebbian learning or spike-timing-dependent plasticity^1,3,4^ are useful to learn representations of features based on the statistics of the input stream^5–7^, they lack a mechanism to incorporate neuromodulatory feedback signals that condition learning on reward, punishment or novelty^8,9^. By contrast, three-factor rules such as reward-modulated spike-timing-dependent plasticity (R-STDP)^10^ not only respond to input statistics but also incorporate an additional modulatory signal, allowing synaptic plasticity to be regulated based on behavioural context. Specifically, three-factor rules rely on Hebbian signals locally available at each synapse combined with a global broadcast signal (third factor)^2,8,9^. Three-factor learning rules are closely linked to reinforcement learning (RL)^10^, a machine learning approach in which artificial agents acquire knowledge by interacting with their surroundings, striving to maximize cumulative rewards^11^, similar to biological agents^12^. Software implementations of deep RL^13,14^ have excelled at complex games, for example, the game of Go^15^, and at navigation-related tasks such as autonomous robots, drones and cars^14,16,17^. However, the computational demands and power consumption of such software-based RL systems remain substantial, particularly when deployed in real-time, resource-constrained environments. Moreover, the necessity of deep RL to rely on the backpropagation algorithm makes it not only biologically implausible^18^ but also difficult to implement in energy-efficient hardware.

Implementing three-factor learning directly in hardware offers an alternative towards more efficient and scalable learning systems^19,20^. Such neuromorphic computing approaches^21–24^ have been realized using both complementary metal–oxide–semiconductor (CMOS) and memristive technologies. Although CMOS-based platforms are more mature in terms of integration^25^, they typically rely on digital architectures with physically separated memory and processing units, resulting in energy and latency bottlenecks in large-scale systems^24^. By contrast, memristors offer unique capabilities such as analogue, tunable synaptic weights and in-memory matrix–vector multiplications via crossbar arrays^26–30^, making them particularly attractive in energy-efficient, bio-inspired computing applications. However, to fully exploit these advantages, it is crucial that as many operations as possible are carried out directly on the memristive hardware itself^31,32^.

So far, only few memristor-based implementations of three-factor learning have been reported^33–36^, all limited to the use of individual devices only. Sarwat et al. implemented reward-based three-factor learning on memristors by combining light and voltage signals^33^. However, as the devices display only digital switching, they could not replicate the analogue nature of biological synapses, preventing fine-grained synaptic modulation essential for three-factor learning. An alternative to this approach is memristor-based circuits implementing R-STDP^34–36^. However, R-STDP has been shown to be less powerful than, for example, temporal difference (TD) learning rules^10,11^, which are optimally suited to navigation tasks^37,38^. Most importantly, an in situ approach in which memristive weights are trained online (that is, trained during runtime^27^) is still lacking.

Here we propose a framework for memristor-based TD learning. Specifically, we use the so-called TD error as a third factor, analogous to the reward prediction error delivered by the neurotransmitter dopamine in the mammalian brain^8,39,40^. The TD error signal is calculated in the critic module of an actor–critic network, which has been proposed as a fundamental architecture for reward-based learning in the brain^41–45^. Notably, and contrary to previous demonstrations of RL on memristors, our combination of a biologically plausible network architecture and three-factor learning enables the execution of all critical operations in hardware. Our memristors (1) serve as synaptic weights that are trained online and in memory; (2) they determine the next actions of the network; and (3) they compute the weight updates associated with the TD error. As such, our approach minimizes external recourse to software and allows full in-memory training, which enhances the processing speed and efficiency of artificial neural networks^46^. We demonstrate the applicability of our framework in two common neuroscience navigation tasks, the T-maze and the Morris water maze^47–49^. Our implementation relies on analogue valence change memories (VCM) consisting of a dielectric HfO_2_ layer combined with a conductive metal oxide (CMO)^50–53^; however, it is independent of the memristor type.

## Results

### Memristor-based actor–critic TD learning

**Fig. 1:**
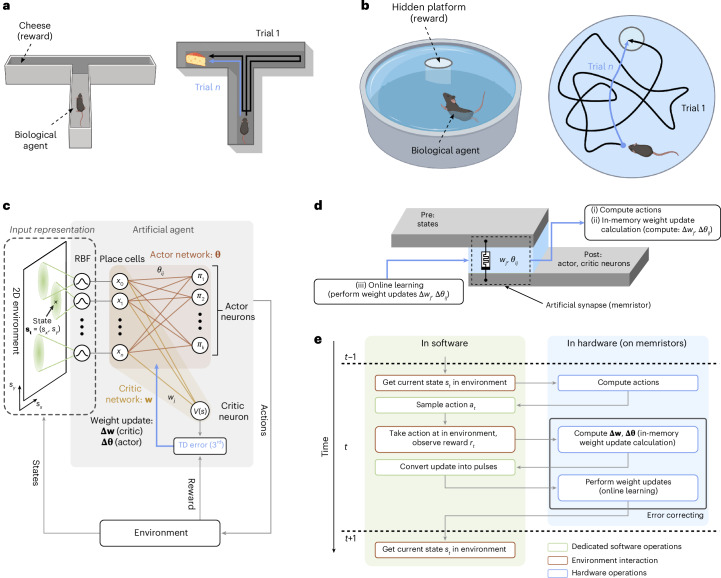
Bio-inspired RL. **a**, Sketch of a T-maze experiment. A biological agent (mouse) is navigating through a maze to find a reward (cheese). The T-maze consists of a stem and two lateral arms, with a reward at the end of one arm. The biological agent is placed at the bottom of the maze and can take a sequence of actions: going forwards or backwards, and, at the junction of the T-maze, turning right or left. Initially, the action choice is random (black trajectory), but the agent learns to choose actions that maximize the future reward (blue trajectory). **b**, Sketch of the Morris water maze experiment. A biological agent (mouse) is placed at a random location in the pool and strives to escape from the cold water onto a hidden platform (reward). The mouse is free to move in any direction. The mouse starts by swimming in circuitous paths (black trajectory) before learning over many trials to approach the platform on a nearly direct path (blue trajectory). **c**, Artificial agent with actor–critic TD learning (schematic). Place cells (state neurons; left) represent the momentary state **s**_**t**_ (position) of the agent and are connected via a weight matrix **θ** to actor neurons, which encode the probabilities *π*_*i*_ for choosing action *i*. The place cells are also connected via the weight vector **w** to a critic neuron representing the value *V*(*s*) of the current state. The TD error (3^rd^) is a reward prediction error, analogous to the dopamine signal in biology^8,11^. It is used to update the weights in the actor–critic network (**w**, **θ**). **d**, Illustration of a memristor used as an artificial synapse in the actor–critic network. Memristors implement both weights **w** and **θ**. They perform online weight updates, compute the actions and are used for the in-memory calculation of the weight updates Δ**w** and Δ**θ**. **e**, Flowchart of the proposed bio-inspired actor–critic RL algorithm split into a software and hardware part. The hardware part employs the memristors to perform all operations highlighted in **d**. The software part mostly emulates the role of the environment by taking steps and returning rewards. Dedicated software calculations are kept minimized and only comprise sampling the actions, calculating the number of voltage update pulses from the corresponding weight updates, and computing the RBFs of the input representation. The combination of an online learning algorithm and continuous in-memory weight update calculation enables error-correcting weight updates as errors caused by the device non-idealities are compensated for in the next learning iteration. Panels **a** and **b** created with BioRender.com.

As a testbed for our bio-inspired three-factor learning framework on memristors, we consider the T-maze and Morris water maze navigation tasks (Fig. 1a,b, respectively). Both mazes are widely used to gain insight into the spatial learning and memory of animals^47–49^. In both experiments, a biological agent (mouse) initially moves randomly (trial 1), but gradually learns to take an efficient (direct) path to a predefined reward (trial *n*). The improvement in the mouse’s spatial navigation, trial by trial, is an example of reward-based learning^37,38^. Here we formulate both navigation tasks in a biologically plausible actor–critic network (Fig. 1c)^42–45^.

The network has two main parts, an actor and a critic component. The critic computes the value of each spatial position or state^11^, which measures its proximity to the reward, whereas the actor network represents the learned action choices. The actor–critic architecture exhibits multiple similarities with the functions of certain brain areas^54–57^ (Supplementary Fig. 1) and with widely used computational neuroscience models^37,38,58,59^. At time *t*, the agent’s position in the two-dimensional environment is given by the state **s**_**t**_ = (*s*_*x*_, *s*_*y*_), where *s*_*x*_ and *s*_*y*_ are Cartesian coordinates. This position is encoded by *n* place cells^60^ with activities (*x*_1_; *x*_2_…*x*_*n*_) = **x**_**t**_. Place cells are implemented as radial basis functions (RBFs) and form a fixed input layer to the actor–critic network (Methods). Given the agent’s position in the place-cell representation, a single actor–critic layer is sufficient to learn complex navigation tasks^61,62^, thereby reducing the size and complexity of typical RL networks.

In our network, the connection from any place cell *j* and the critic neuron(s) (a single one in our model) is characterized by a weight *w*_*j*_. The activity of the critic neuron *V*(*s*_*t*_) = ∑_*j*_*w*_*j*_*x*_*j*_ assigns a value *V*(*s*_*t*_) to each state *s*_*t*_. In the actor network, each neuron *i* encodes a different action *a*_*i*_, such as moving forwards, left or right (Methods provides details on the action selection). The weights, labelled *θ*_*ij*_, connect the place cells *j* (pre-synaptic neurons) to the actions *i* (post-synaptic neurons). The weights of both networks are adjusted by a neo-Hebbian three-factor rule^2,9,63,64^, which depends on the activity of the pre-synaptic (*j*) and post-synaptic (*i*) neuron via a ‘Hebbian’ coincidence detection ‘*H*’ (Methods) and a third factor called 3^*rd*^:

1 $$\Delta {\theta }_{ij}=\alpha \times {3}^{rd}\times {H}^{act}(i,j),\,\Delta {w}_{j}=\alpha \times {3}^{rd}\times {H}^{cri}(j),$$

where *α* is a learning rate parameter and the superscripts act and cri refer to actor and critic, respectively. The third factor 3^*rd*^ is related to phasic dopamine signals in the brain^8,54^ and represents the TD error *δ*_*t*_ (ref. ^11^):

2 $${3}^{rd}={\delta }_{t}=r({s}_{t})+\gamma \times V({s}_{t+1})-V({s}_{t}).$$

Here *r*(*s*_*t*_) is the reward received at time *t*, the parameter *γ* ≤ 1 is a discount factor, *V*(*s*_*t*_) is the value of the state *s* at time *t* and *V*(*s*_*t*+1_) is the value of the next state at time *t* + 1 after an action has been taken. The discount factor influences the relative importance of distant to immediate rewards^13^. This architecture and learning rule differ from a previously reported memristor-based actor–critic network^65^ employing backpropagation and gradient descent algorithms—two less biologically plausible techniques than those implemented here^18^.

In our actor–critic learning scheme, analogue memristors act as the artificial synapses *θ*_*ij*_ and *w*_*j*_ (Fig. 1d). The memristor weights are not only employed to statically encode the learned actions and values but they also (i) compute the actions, (ii) determine the weight updates based on the TD error (in-memory weight update calculation) and (iii) are updated in an online fashion according to equation (1) (online learning). Importantly, the same memristors are employed to perform all these tasks, highlighting their multifunctional role. Moreover, our implementation does not require any weight read-outs, which reduces data movement. The flowchart in Fig. 1e summarizes a single time step of the algorithm, segmented into its software and hardware components. Some operations are still performed in software but are limited to the interaction of the agent with its environment, a few computationally inexpensive operations (that is, action sampling, conversion of weight update into pulses) and the evaluation of RBFs to obtain place-cell representations.

Executing the majority of the algorithm in memory, as here, is known to enhance the processing speed and lower the energy consumption of learning processes on crossbar arrays^46^. By contrast, conventional RL implementations on memristive hardware only partially exploit in-memory computing. Typically, (1) the action and the weight update computation—both complex operations—are carried out in software and (2) the memristor weights are continuously read-out in case of write–verify schemes^66–68^. Supplementary Table 1 compares our TD learning hardware implementation with previous demonstrations of RL algorithms on memristors. As the TD error is calculated at each step, errors due to imperfect weight updates on memristors are trained away in the next iteration, providing error-correcting capabilities. With our online learning strategy, the errors are automatically incorporated into the in-memory weight update calculation. The learning loop is detailed in the ‘In-memory learning loop’ section.

### Analogue memristor synapses as active components of actor–critic networks

**Fig. 2:**
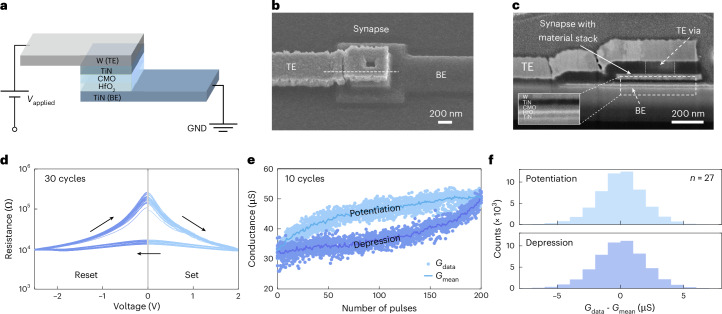
Fabrication, characterization and analysis of the analogue, VCM-type memristors used in this study. **a**, Schematic of the material composition and biasing scheme. The active layers consist of HfO_2_ and a CMO. Our memristors are operated by grounding the TiN bottom electrode (BE) and applying an electrical signal to the W top electrode (TE). A conductive filament (CF) is initially created during a forming step by applying a negative voltage at the TE (Supplementary Fig. 2). **b**, Scanning electron microscopy image providing an angled view of the vertical VCM stack. The active area of the artificial synapse is given by the overlap of the top and bottom electrodes. **c**, Focused-ion-beam (FIB) cross-section image cut along the dotted line shown in **b**. The inset shows the deposited material stack including the active HfO_2_–CMO bilayer of the VCM memristor. **d**, Resistance–voltage (*R*–*V*) characteristics displaying the resistive switching behaviour of the fabricated devices. They exhibit reproducible switching with gradual and quasi-symmetrical set and reset processes. **e**, Dynamic measurements displaying the synaptic potentiation and depression curves as a function of the number of applied pulses. Data from ten distinct cycles, along with the mean, are shown. Identical pulse trains were used to perform this measurement: 200 set pulses at 2.5 V with a 1.5-μs pulse width for potentiation and 200 reset pulses at –2.7 V with a 10-μs pulse width for depression. The pulse parameters (amplitude and duration) were specifically chosen to achieve a good compromise between the linearity of potentiation/depression and the noise in weight updates. **f**, Histograms showing the deviations of both potentiation and depression measurements from their respective means for 27 distinct devices.

Our actor–critic framework relies on analogue memristors as hardware components consisting of a W/TiN/CMO/HfO_2_/TiN stack and operating on the VCM effect (Fig. 2a). Both HfO_2_ and CMO layers are involved in the switching process: a conductive filament grows through the HfO_2_ layer, whereas the CMO acts as an oxygen reservoir layer^52,69,70^. This CMO–HfO_2_ bilayer film offers better analogue switching characteristics than Ti–HfO_2_ stacks^52^.

**Extended Data Fig. 1:**
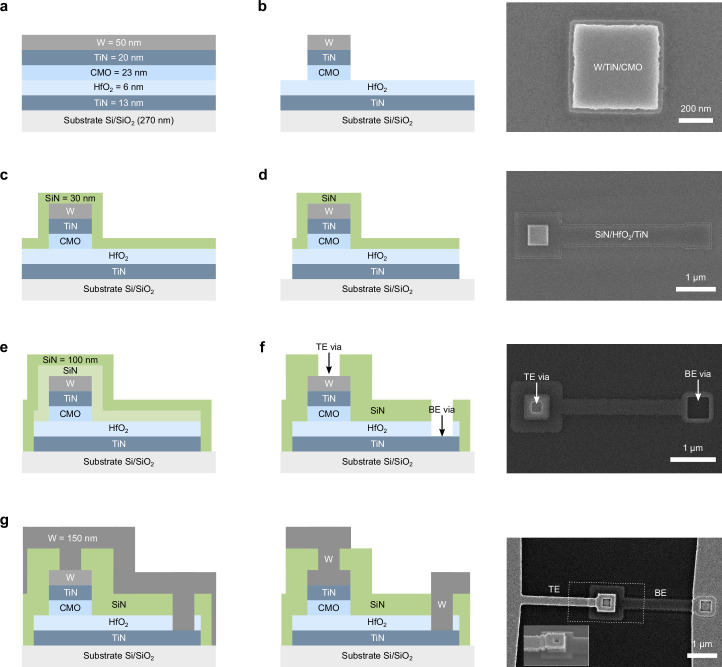
Detailed fabrication flow of the analogue memristors with Scanning Electron Microscopy (SEM) pictures. (**a**) Deposition of the CMOS- and BEOL-compatible layers on a Si/SiO_2_ substrate. The TiN and HfO_2_ layers are deposited by a Plasma-Enhanced Atomic Layer Deposition (PEALD) process at 300 ^∘^C, without breaking the vacuum to prevent oxidation of the TiN. The Conductive Metal Oxide (CMO), TiN and W layers are then deposited by sputtering. During the transfer from the ALD chamber to the sputtering system, the layer stack experiences a brief exposure to the atmosphere, during which moisture and protons may be incorporated into the device stack^73^. (**b**) Etching of the top three layers. The etch defines the active area. (**c**) Encapsulation of the active area by SiN. This material is deposited via Plasma-Enhanced Chemical Vapour Deposition (PECVD) process at 300 ^∘^C. (**d**) Etching of the bottom two layers. The HfO_2_ and TiN are etched to define the bottom electrode (BE). (**e**) Encapsulation of the BE by SiN. (**f**) Via opening to provide access to the memristor device. For the top electrode (TE) via, SiN is etched, while for the bottom electrode (BE), both SiN and HfO_2_ are etched. (**g**) Sputtering and patterning of a W TE. A SEM picture of the final device is shown. The inset provides an angled view.

The fabrication procedure, which employs processes compatible with CMOS and back-end-of-line (BEOL) technologies, closely follows the method presented in ref. ^52^, differing only by the use of electron-beam lithography instead of optical lithography steps. A detailed description of the fabrication process is provided in Extended Data Fig. 1. Our memristors have an active area of 600 × 600 nm^2^ (Fig. 2b). The focused-ion-beam cross-section image (Fig. 2c) displays the deposited material stack.

Under direct current (d.c.) operation, the HfO_2_–CMO bilayer exhibits reproducible resistive switching properties, as demonstrated by the resistance–voltage (*R*–*V*) characteristics (Fig. 2d). Both transitions from the high-resistance state to the low-resistance state and from the low-resistance state to the high-resistance state are gradual. Such a behaviour originates from the modulation of oxygen vacancies within the CMO layer, and from the confinement of the electric field and temperature within the HfO_2_–CMO bilayer^71^. This confinement, in turn, enables controlled and continuous changes in electrical conductivity^52,72^. We also investigated synaptic potentiation and depression by stimulating our devices with identical pulse trains. Figure 2e reports ten full potentiation/depression cycles together with their mean values. The measurement data demonstrate a gradual and controlled switching during pulsed operations, resulting in multiple reproducible non-volatile states that represent the actor (*θ*_*ij*_) and critic (*w*_*j*_) weights in our actor–critic network (Fig. 1c).

We quantified the noise level in the potentiation and depression curves for multiple devices by subtracting the measurement data from their respective means (Fig. 2f). The obtained histogram serves as a measure of the update noise within our devices. The noise can be attributed to the read operation^68^, cycle-to-cycle variability and the measurement setup (Methods and Supplementary Fig. 3). Although protons and water molecules could be incorporated during layer deposition and are known to play a crucial role in the switching operation^73^, no detailed analysis was performed, as endurance measurements (not shown here) demonstrated reliable operation up to 10^8^ programming cycles. The same applies to interfacial reactions, such as the possible oxidation of the TiN electrodes.

### In-memory learning loop

**Fig. 3:**
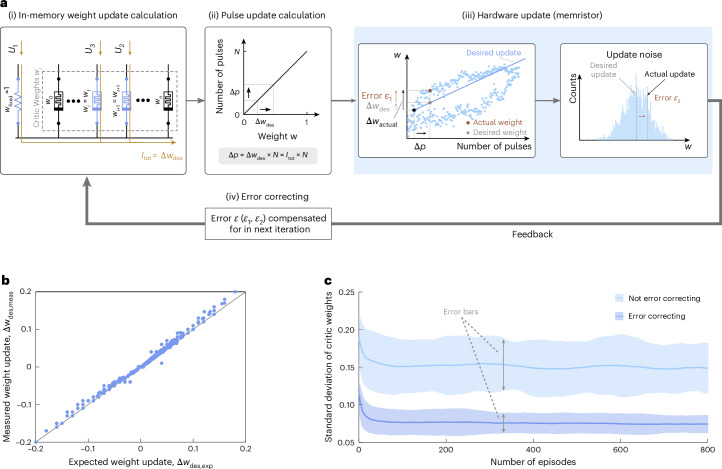
In-memory learning loop. **a**, The learning procedure starts with the in-memory calculation of the desired weight update Δ*w*_des_ through a subnetwork including three weights *w*_fixed_, *w*_*t*+1_ and *w*_*t*_. On the basis of the current location of the agent, the voltages *U*_3_ and *U*_2_ are applied to the two critic devices storing *w*_*t*_ = *V*(*s*_*t*_) and *w*_*t*+1_ = *V*(*s*_*t*+1_). The value of Δ*w*_des_ is then translated into the corresponding number Δ*p* of update pulses, followed by the actual weight update on the memristor *w*_*t*_. Two sources of errors are introduced during the update: an error *ϵ*_1_ because of the nonlinear dependence of the weight update on the number Δ*p* of applied voltage pulses and an error *ϵ*_2_ because of the inherent noise in the memristor updates. During the subsequent iteration, both error terms are taken into account and, therefore, compensated through the in-memory calculation of the next desired weight update (feedback). The update loop, thus, has capabilities to correct errors. **b**, Experimentally measured in-memory weight updates (Δ*w*_des,meas_) as a function of the expected software weight updates (Δ*w*_des,exp_). Δ*w*_des,meas_ were measured out of the subnetwork in **a**, for various weights *w*_*t*_ and *w*_*t*+1_, unit-free values within the range of 0–1, whereas *w*_fixed_ has a fixed value of 1. Δ*w*_des,exp_ was calculated with equation (3) using the values *V*(*s*_*t*+1_) and *V*(*s*_*t*_), within the range of 0–1, whereas *r*(*s*_*t*_) has a fixed value of 1. The measured values of Δ*w*_des_ are in good agreement with the calculated values, with their absolute difference never exceeding 0.03 (or 3%). Note that for both experiments and theory, a value of *α* = 0.2 was used, which, according to equation (3), implies that the weight updates cannot be larger than 0.2. **c**, Mean and error bars for the standard deviation of critic weights are compared between the cases with and without the error-correcting feedback as a function of the episode number. The results are extracted from 1,000 distinct simulated runs using an actor–critic RL scenario. The plot highlights the impact of the error correction mechanism on the variability of the learned weights.

Our actor–critic RL framework supports learning, that is, the adaptation of *θ*_*ij*_ and *w*_*j*_, on both hardware and in-software-emulated memristors. The training of critic weights (actor weights work analogously) is depicted in Fig. 3a in the case of one-hot encoding: only one place cell is active at a time (Supplementary Note 1), which is effective if the state space is limited.

The learning process begins with the calculation of the desired weight update in hardware (Fig. 3a(i)), denoted as Δ*w*_des_. This is done in memory by performing an in situ vector–vector multiplication using two critic memristors (*w*_*i*_ and *w*_*i*+1_) along with a fixed-value resistor (*w*_fixed_) that has a weight equal to 1. Since in one-hot encoding, each critic weight stores the value *V*(*s*) of one state, *w*_*i*_ and *w*_*i*+1_ are chosen such that they correspond to *V*(*s*_*t*_) (current state) and *V*(*s*_*t*+1_) (next state) and, thus, labelled *w*_*t*_ and *w*_*t*+1_, respectively. In other words, the same memristors store the critic weights and are used to calculate the weight update. By applying the voltages *U*_1_ = *α* × *r*(*s*_*t*_), *U*_2_ = *α* × *γ* and *U*_3_ = −*α*, to *w*_fixed_, *w*_*t*+1_ and *w*_*t*_, respectively, the desired weight update is obtained for the memristor storing *w*_*t*_:

3 $$\Delta {w}_{des}=\alpha\times r({s}_{t})+\alpha \times \gamma \times V({s}_{t+1})-\alpha \times V({s}_{t}).$$

The update is determined by measuring the total output current *I*_tot_ resulting from the vector–vector multiplication. The detailed derivation is provided in the Methods and Supplementary Note 2, whereas the general case of beyond one-hot encoding with higher-dimensional input activations is discussed in Supplementary Note 7.

In the second part of the learning algorithm (Fig. 3a(ii)), the desired weight update Δ*w*_des_ is converted into the number of update pulses Δ*p* = Δ*w*_des_ × *N*, assuming a linear potentiation and depression of the memristor conductance. Here Δ*w*_des_ corresponds to the extracted output current *I*_tot_ = Δ*w*_des_, and *N* is the total number of pulses, which is a constant (200 in our case). This conversion is currently done in software, but could be directly implemented on chip^24,74,75^.

**Extended Data Fig. 2:**
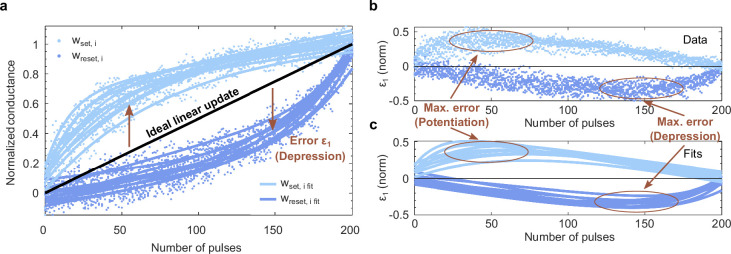
Estimation of the error *ϵ*_1_ arising from the difference between idealised (perfectly linear) and actual (non-linear) weight updates. (**a**) Measured and fitted potentiation and depression curves of ten different cycles from a single memristor device. As mentioned in the main text, an error term *ϵ*_1_ is introduced due to the difference between the ideal (linear) potentiation/depression (black line) and non-ideal measurement curves (dark and light blue). This error *ϵ*_1_ is extracted by subtracting the measurement data (**b**) and the fits (**c**) from the ideal linear curve for all ten cycles. In both cases, the error is directly related to the non-linearity of the potentiation/depression and is largest at the beginning of the potentiation (light blue) and depression curves (dark blue), as indicated by the red circles. This implies that weight updates when the memristor conductance is close to the beginning of the potentiation and depression curves result in a larger error *ϵ*_1_. Because the weights in our actor-critic reinforcement learning tasks are generally more often increased than decreased (their initial value is zero), the problem is more severe in the case of potentiation than depression (see for example Fig. 4b in the main text). The first update of each weight is therefore the most impacted one.

As the third step of the learning loop, the conductance of the targeted memristor is updated by applying Δ*p* pulses (Fig. 3a(iii)). The assumed linear ‘Δ*w* versus Δ*p*’ relationship during the calculation of Δ*p* introduces an update error *ϵ*_1_ because of the nonlinearity of our memristors’ potentiation/depression curves (Fig. 2e). This error *ϵ*_1_ is particularly pronounced near the devices’ minimum and maximum conductance values (saturation regions), where deviations from linear weight updates are the largest (Extended Data Fig. 2). A second error *ϵ*_2_ arises because of update noise, as introduced in Fig. 2f. Both errors are automatically taken into account and, therefore, compensated for by the in-memory weight update calculation in the next iteration of the learning loop (Fig. 3a(iv)), leading to an error correction mechanism (Methods and Supplementary Note 4). The learning loop for in-software-emulated memristors is discussed in Supplementary Note 3.

Although *ϵ*_1_ impacts the weight update, the assumption of a linear update offers substantial advantages over more complex schemes^76^. In particular, it avoids read-outs of the current weights to determine the position within the potentiation/depression model curves and eliminates the need to store the true weight update curves of each memristor. Hence, faster and more energy-efficient in situ weight update processes are possible^27^.

The calculation of Δ*w*_des_ in hardware assumes a linear ‘resistance versus voltage’ relationship and includes the measurement noise. We, thus, empirically verify the accuracy of the in-memory weight update evaluation through a comparison between the measured (Δ*w*_des,meas_) and expected (Δ*w*_des,exp_) weight updates (Fig. 3b) for different weight combinations of the two memristors involved, namely, *w*_*t*_ and *w*_*t*+1_. In all combinations tested, we found good agreement between the experimental measurements and theoretical expectations, with an error below 3%.

To further examine the error-correcting properties of our approach, we consider the standard deviation of the trained critic weights across 1,000 independent training runs in a simulated actor–critic RL task (Fig. 3c; Methods). The incorporation of the error terms *ϵ*_1_ and *ϵ*_2_ into the calculation of the desired weight update is responsible for the error-correcting feedback loop. This results in reduced variability in the learned weights, as indicated by the lower standard deviation compared with the case in which the error terms are not compensated for: the errors do not accumulate over several episodes, but are trained away at every iteration. Furthermore, with error correction, the error bars become narrower, corresponding to a smaller spread in the weights.

### Learning in discrete space using analogue memristors

The aforementioned analogue memristors are used to solve the T-maze navigation task (Fig. 1a) in discrete space. It involves an agent navigating through the maze and adapting its policy to locate a reward. The environment consists of nine states labelled 0–8 with the reward located in the left corner of the T-maze (state 6). The limited state space allows for the use of one-hot encoding to represent the agent’s current position in the maze. This means that only one place cell in Fig. 1c is active at a time, which directly corresponds to the agent’s location. This encoding eliminates the need for RBFs, and the activation **x**_**t**_ is equal to the state **s**_**t**_. Each place cell is then connected to two actor neurons that encode the possible actions and one critic neuron that computes a value estimate of the current location. The actions can either be ‘moving forwards/backwards’ (all states except state 4) or ‘moving left/right’ (state 4).

**Fig. 4:**
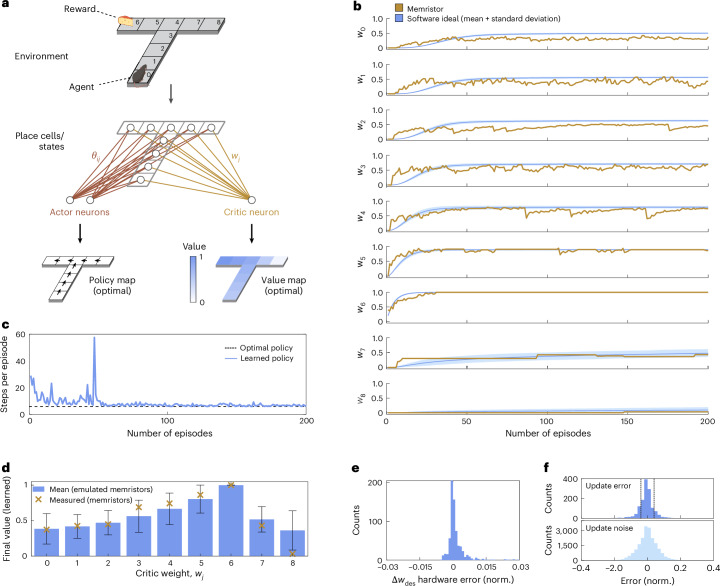
Bio-inspired actor–critic RL demonstrated on a proof-of-concept T-maze navigation task. **a**, An agent must navigate through a T-maze environment made of nine states labelled 0–8 to reach a reward (state 6). Place cells represent the position of the agent and each of them corresponds to a distinct state in the environment (one-hot encoded). Two actor neurons encode forwards/backwards (all states except 4) or left/right movements (state 4). The single critic neuron computes an estimate of the value of a given state. The ideal policy and value maps are illustrated at the bottom. The synaptic weights connecting place cells to actor neurons (*θ*_*ij*_) and place cells to the critic neuron (*w*_*j*_) are implemented by memristors. For each run, two out of the nine critic memristors are implemented in hardware, whereas the behaviour of the other critic and all actor weights is emulated in software. Therefore, five distinct configurations were investigated to measure all critic weights in hardware: (*w*_0_, *w*_1_), (*w*_2_, *w*_3_), (*w*_4_, *w*_5_), *w*_6_ and (*w*_7_, *w*_8_), where each *w*_*j*_ corresponds to a distinct device. The hardware memristors are updated in an online manner and perform the in-memory calculation of the desired update Δ*w*_des_. **b**, Measured critic weights *w*_0_–*w*_8_ (orange) over 200 episodes compared with the ideal software case (blue). The software curves correspond to the average of 1,000 distinct runs, with light blue indicating two standard deviations^101^. Each critic weight was implemented by a different memristor. **c**, Number of steps per episode required to reach the reward as a function of the episode number, extracted as the mean value from all the configurations shown in **b**. The learned policy approaches the optimal one (six steps between the initial position and the reward). **d**, Mean and error bars of the critic weights after 200 episodes for runs with in-software-emulated memristors (blue) compared with the experimental values (yellow crosses). The values of emulation are the average of 1,000 distinct runs, with the error bars indicating two standard deviations. **e**, Histogram showing the error between the measured (Δ*w*_des,meas_) and expected (Δ*w*_des,exp_) theoretical values for the in-memory calculation of the weight update. Δ*w*_des,meas_ is obtained using equation (8) (Methods). **f**, Comparison between the experimental update error (top) and the update noise (bottom) extracted from the corresponding potentiation and depression curves. The update error is smaller or equal to the extracted update noise. The resulting accuracy allows for an exact tuning of the analogue memristor weights, thereby confirming the feasibility of in-memory/online learning. The dashed lines in the top histogram denote the hardware error limits shown in **e**. Panel **a** created with BioRender.com.

The concepts of online learning, in-memory weight update calculation and error correction presented in Fig. 3 are combined to realize bio-inspired learning in an actor–critic network. The latter comprises 27 synaptic weights, including 9 × 2 = 18 actor weights (*θ*_*ij*_) and 9 × 1 = 9 critic weights (*w*_*j*_). Each of these weights is implemented by a different hardware memristor (Methods). For every run, two out of the nine critic weights are represented by physical memristors and updated in hardware via online training. The same two hardware devices additionally implement the in-memory weight update calculation introduced in Fig. 3a. The outcome is the learned policy and the corresponding value map (Fig. 4a).

The measurements of the learned critic weights *w*_0_–*w*_8_ as a function of the episode number are displayed in Fig. 4b, together with the software runs that use ideal synaptic weights (continuous, linear and no noise). The measured curves follow the software runs, with the agreement being better for critic weights associated with states closer to the reward. Deviations between the measurements and ideal runs can be attributed to fluctuations related to the nonlinear potentiation/depression curves (error term *ϵ*_1_) and update noise (error term *ϵ*_2_). This non-ideal behaviour is the most evident at the start of training and for weights that remain small during training (that is, *w*_0_ – *w*_2_), where *ϵ*_1_ is the most pronounced. However, since these errors are fed back into the in-memory weight update calculation in subsequent iterations, they are gradually corrected over time.

**Extended Data Fig. 3:**
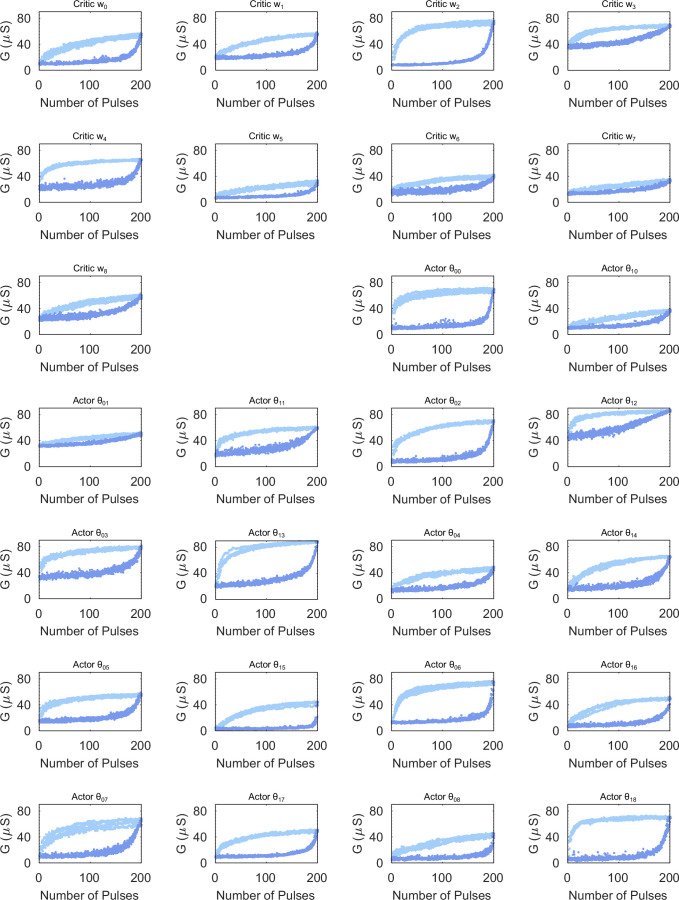
Measured potentiation and depression curves of the memristor devices used for the hardware runs of the T-maze navigation task (Fig. 4 of the main text and Supplementary Note 7). The 9 critic weights are denoted as *w*_*j*_ and the 18 actor weights as *θ*_*ij*_.

We also tested the implementation of all actor weights in hardware using 18 different memristors (Supplementary Note 6). Extended Data Figure 3 presents the potentiation and depression curves of the memristors utilized for the hardware critic and actor weights. Overall, in-memory training, including online weight updates and weight update calculations, consumes 28.2 μJ of energy (Supplementary Note 8).

The agent initially finds the reward through random exploration, subsequently through the reinforcement of successful trajectories. With an increasing number of episodes, the agent predominantly exploits the stereotypical trajectory it has learned to reach the target. This behaviour can also be observed in Fig. 4c, which illustrates the number of steps required to reach the reward as a function of the episode number. At the beginning of the learning experiment, the number of steps is high because the actor weights are still small, resulting in random action choices. As the learning experiment progresses, the number of steps converges to the optimal trajectory of six steps (Fig. 4c, black dotted line). Even after the correct path has been learned (that is, after around 50 episodes), the finite temperature of the softmax action selection ensures continued exploration via random actions, thereby addressing the exploration–exploitation dilemma (Supplementary Fig. 4)^11^ and inducing slight fluctuations in the number of steps.

The values of the trained critic weights after the last episode are shown in Fig. 4d. The experimental data are compared with results from runs using only in-software-emulated memristors. All measurements fall within the error bars of the simulated values, proving that the in-software-emulated memristors closely replicate the learning of their physical counterparts.

Importantly, in our measured runs, the weight updates (Δ*w*_des_) are directly calculated in hardware, allowing for the entire learning loop to remain in memory. Hence, the algorithm operates without explicit read-outs of the weight values at any point, minimizing off-device computations. We only measured the critic weights for visualization purposes during training.

The in-memory weight update calculations introduce only minimal deviations from the targeted values (Fig. 4e), which reports the difference between the measured (Δ*w*_des,meas_) and expected (Δ*w*_des,exp_) in-memory weight update values. The absolute error stays below 0.04, consistent with the reference measurement shown in Fig. 3b. It becomes even more evident that the error in Δ*w*_des_ is small, when compared with its counterpart introduced by the memristor weight update processes (Fig. 4f, top). Finally, we relate this update error to the overall update noise (Fig. 4f, bottom). The latter is extracted from the corresponding potentiation and depression characteristics of the measured memristors. The update error is smaller than or equal to the extracted update noise, suggesting that weight updates within the analogue programming process of our devices are very accurate, establishing the feasibility of online learning for our memristors.

### Learning in continuous space using in-software-emulated memristors

**Fig. 5:**
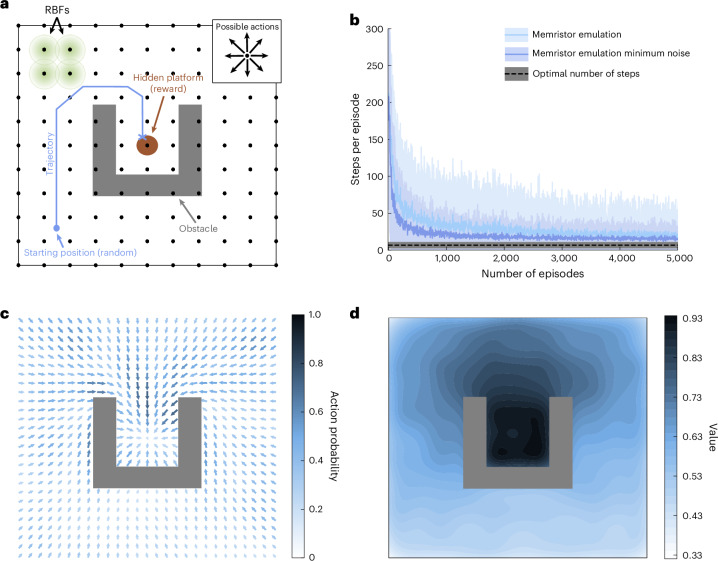
Actor–critic framework applied to a navigation task akin to the Morris water maze using in-software-emulated memristors. **a**, Illustration of the task objective. An artificial agent is placed at a random location in the maze (starting position; blue) and navigates through the maze (trajectory; blue) until it reaches the reward (hidden platform; red). To make the task more challenging, a U-shaped obstacle (grey) forces the agent to reach the reward from above^10^. For each step in the maze, the agent can select one out of eight actions (inset). The two-dimensional continuous state space is mapped to a grid of 11 × 11 overlapping place cells (black dots denote the centre of place cells). The closer the location of the agent to a place cell, the larger the input activation, given by Gaussian RBFs (green circles correspond to one standard deviation of the Gaussian function). **b**, Mean and standard deviation of the number of steps per episodes for 100 random seeds learned on in-software-emulated memristors (light blue) compared with the case in which the update noise is set to the minimum of all memristors (dark blue). The black dotted line indicates the mean and standard deviation achievable by a noiseless, ideal learner. Note that a random walk policy would take the agent 450 steps on average to find the reward. **c**, Mean policy map after training on in-software-emulated memristors. Each arrow points towards the average direction taken by the agent. The colour and length of these arrows are scaled according to the probability of taking the shown direction. From any initial position, actions leading to the target area are learned with increasing probabilities closer to the reward, as expected from ref. ^10^. **d**, Mean state value map after training. The closer the value is to the reward area, the higher is the learned value.

To demonstrate that our framework can be applied to more complex problems and scaled up to larger memristor arrays, we set up the two-dimensional Morris water maze experiment (Fig. 1b)^10,37,38,47^ and perform simulations exclusively on the previously validated in-software-emulated memristors (Methods). In this task, the agent is randomly placed at a starting location in a water maze. Its goal consists of reaching a reward in the form of a hidden platform (Fig. 5a). Once this happens, a reward signal is released and the episode ends.

**Extended Data Fig. 4:**
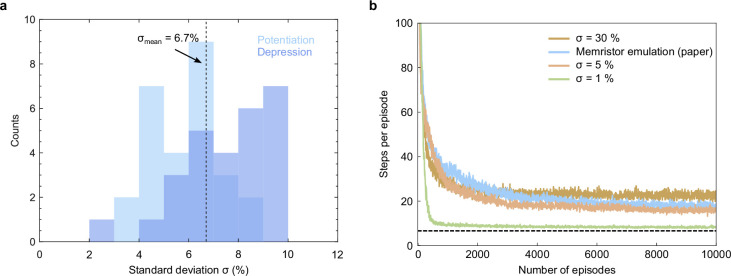
Effect of the noise level on the convergence speed of the Morris water maze navigation task. (**a**) Extracted update noise for both potentiation and depression curves of all in-software-emulated memristors. A single parameter *σ*, corresponding to the standard deviation of all 10 overlapped potentiation or depression measurement curves was extracted for each measured device. (**b**) Number of steps per episode plotted against the number of episodes for different noise levels, compared to the memristor emulation in the paper. The curves show the mean of 100 distinct simulation runs with an applied running average of 10 episodes to improve comparability between the runs. The in-software-emulated memristors exhibit noise levels between 2.9% and 10%, with a mean of 6.7%.

**Extended Data Fig. 5:**
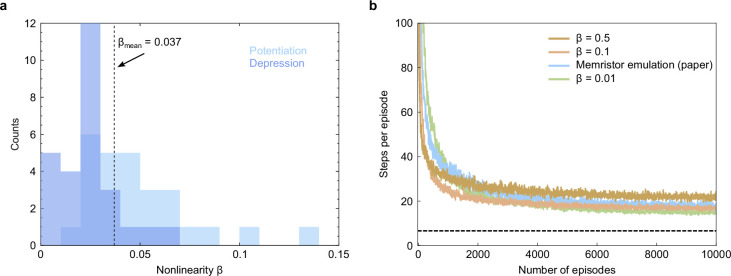
Effect of non-linearity on the convergence speed of the Morris water maze navigation task. (**a**) Extracted non-linearity parameter *β* for both potentiation and depression curves from our 27 experimental devices, used in the corresponding software models. (**b**) Number of steps per episode plotted against the number of episodes for different non-linearity parameters *β*, compared to the memristor emulation in the paper. Each curve represents the average of 100 independent simulation runs, with an applied running average of 10 episodes to improve comparability between the runs.

Figure 5b displays the number of required steps to reach the reward versus the episode number. Despite the presence of initially large fluctuations probably caused by the update error of the in-software-emulated memristors, the number of steps gradually converges to a mean value close to its optimum of around 6.5 steps obtained through software runs (black dotted line). We attribute this noise resilience to the error correction mechanism of our approach. Reducing the update noise of each device to the minimum value among all 27 memristors, while keeping the same potentiation/depression curves, allows the agent to more rapidly attain the reward and drastically reduce the number of episodes for convergence (Fig. 5b, dark blue line). A VCM technology with better controlled noise level, such as that demonstrated in ref. ^77^, would, therefore, lead to a faster convergence of the learning process, as indicated by the simulation results shown in Extended Data Fig. 4. Moreover, additional simulations revealed that more linear weight update curves are beneficial (Extended Data Fig. 5 and Supplementary Fig. 5).

To gain further insight into the learning process of in-software-emulated memristors, we examine their synaptic weights for the actor (policy map) and critic (value map) networks after training. Figure 5c displays the mean policy map at every possible position of the agent. The vectors represent the most likely action to be chosen at each location in the maze, whereas their length and colour correspond to the probability of that action. Regardless of the starting position, the learned actions lead the agent directly towards the reward. As expected, the action probability is higher for positions close to the reward^10^. Although the maze is symmetric, the action vector field shows slight asymmetries, which are attributed to the nonlinearity of the weight update curves and to the update noise. The corresponding state values at each position are plotted as a colour map (Fig. 5d).

These software results indicate that our framework can potentially be implemented on a physical VCM crossbar array to perform the Morris water maze navigation task. A possible realization is proposed in Supplementary Note 7. Its energy consumption for operations executed on memristors is estimated to be 20 times (39 times) lower than that of a standard memristor (GPU) RL implementation using the backpropagation algorithm (Extended Data Table 1 and the ‘Energy consumption and latency estimation of a crossbar-level implementation’ section).

## Discussion

We implemented actor–critic TD learning on analogue memristors to mimic biological reward-based learning. In contrast to other memristor-based systems (Methods and Supplementary Table 1), our approach takes advantage of a local three-factor learning rule, which allows for full in-memory training with memristors acting as synaptic weights and directly calculating weight updates. We demonstrated learning on our analogue computing platform by first implementing the T-maze navigation task. For that purpose, we made use of the controllable and gradual switching properties of our devices and showed that the learned weights are close to the ideal values obtained in software. A quantification of the error in the in-memory weight update calculation proved that our approach is highly accurate. Although our framework was tested with HfO_2_–CMO bilayer memristors, it is compatible with any memristive hardware. As an outlook for future work, we applied our framework to a complex biologically plausible learning task with a two-dimensional continuous state space, inspired by the Morris water maze experiment. Overall, our results lay the groundwork for full in-memory operations of neuromorphic chips and for the realization of computing engines that are built similar to their biological counterparts. For example, we envision that our framework could be utilized for real-time navigation in autonomous robots. First, our memristors should be integrated into crossbar arrays, allowing for larger-scale demonstrations of TD learning, where all actor and critic weights are stored and updated simultaneously in hardware. Our scheme could also be extended with eligibility traces^9,11^, a biologically inspired technique to accelerate the convergence time of RL experiments.

## Methods

### Place cells

The position of the RL agent is encoded by *n* place cells^60^ with activities (*x*_1_; *x*_2_…*x*_*n*_) = **x**_**t**_, which serve as the input layer to the actor–critic network shown in Fig. 1c. Their construction and functionality are described elsewhere^60,78^. We adopt the same principles here to encode the spatial information. Specifically, in continuous spatial environments, an effective input representation of the environment is achieved through a fixed layer using RBFs, where each place cell is active in a specific region. The use of place cells is instrumental in reducing the size and complexity of actor–critic networks. Given the position of the agent in the place-cell representation, a single subsequent layer is sufficient to learn complex navigation tasks. This contrasts with deep RL networks, which require the training of potentially many hidden layers to achieve useful input representations^79^.

### Action selection and Hebbian term

The actor network assigns a synaptic weight *θ*_*ij*_ to the connection of place cells *j* (pre-synaptic) to the actor neurons *i* (post-synaptic), where each neuron *i* represents a different action *a*_*i*_. The activity of an action neuron *i* is given by

4 $${h}_{i}=\sum _{j}{\theta }_{ij}{x}_{j},$$

where *x*_*j*_ denotes the pre-synaptic activity (that is, activity of the place cells). *h*_*i*_ determines the probability of selecting action *a*_*i*_ in the momentary state *s*_*t*_ through the softmax policy *π*(*i*∣*s*_*t*_) (ref. ^11^):

5 $$\pi (i| {s}_{t})=\frac{\exp ({h}_{i}/T)}{{\sum }_{k}\exp ({h}_{k}/T)},$$

where *k* is the number of possible actions and *T* is the softmax temperature parameter. The latter determines the balance between exploration (executing random actions) and exploitation (application of the learned actions)^11^, with a higher value resulting in increased exploration. In our actor–critic framework, actions are dynamically learned and become increasingly more certain over time as the actor weights grow. Together with the temperature parameter, which ensures continued exploration, even if the actor network favours a particular action, these two mechanisms help prevent the overexploitation of suboptimal trajectories.

The Hebbian term *H*(*i*, *j*) in equation (1) is a combination of signals that are locally available to the synapse, namely, the pre-synaptic activity *x*_*j*_ and the post-synaptic activity *h*_*i*_. It is defined in our model as

6 $${H}^{act}(i,j)=\left\{\begin{array}{ll}(1-{h}_{i}){x}_{j}(t),\quad &\,\text{for}\,\,{i}^{* }=i\\ -{h}_{i}{x}_{j}(t),\quad &\,\text{for}\,\,{i}^{* }\ne i\end{array}\right.,\,{H}^{cri}(j)={x}_{j}(t),$$

where i* is the post-synaptic action neuron that fired following the chosen action.

### Experimental setups

The d.c. characterization of the memristors were performed with the B2912A source measure unit from Keysight. The bottom electrode (TiN) was grounded, whereas the top electrode (W) was biased with a positive or negative voltage. Neither current compliance nor external series resistor was used during the d.c. measurements as the current passing through the device was self-limited by the active layers of the memristor. The electrical measurements of the dynamic characterization were conducted using the 33500B arbitrary waveform generator from Keysight in combination with the RTE1102 oscilloscope from Rhode & Schwarz and a 10-kΩ series resistance. The conductance states of the potentiation and depression curves were determined via the voltage drop across the resistor. For the hardware weight update calculation and the weight updates, two 33500B arbitrary waveform generators from Keysight were combined with the DHPCA-100 amplifier from FEMTO and the RTE1102 oscilloscope from Rhode & Schwarz. More details about the different experimental setups are given in Supplementary Figs. 3 and 7. All the memristor weight updates were performed using identical pulses: 2.5 V with 1.5-μs width for potentiation, and –2.7 V with 10-μs width for depression, with 200 pulses spanning the full conductance range.

### Derivation of the in-memory weight update calculation

The formulas for the in-memory weight update calculation used in the T-maze task are summarized in this section. The learning rule for the critic weight can be rewritten as a scalar product:

7 $$\begin{array}{l}\Delta w({s}_{t})=\alpha \times {3}^{rd}\times {H}^{cri}(j)\\=\alpha \times \left(r({s}_{t})+\gamma \times V({s}_{t+1})-V({s}_{t})\right)=\left(\begin{array}{c}\alpha \times r({s}_{t})\\ \alpha \times \gamma \\ -\alpha \end{array}\right)\cdot \left(\begin{array}{c}1\\ V({s}_{t+1})\\ V({s}_{t})\end{array}\right)\end{array}$$

Here *α* represents the learning rate, *r*(*s*_*t*_) is the reward at state *s*_*t*_, *γ* is the discount factor, *V*(*s*_*t*+1_) and *V*(*s*_*t*_) are the value estimates and *H*^cri^(*j*) is the Hebbian term of the critic. The latter is equal to 1 (that is, *H*^cri^(*j*) = 1) in the case of one-hot encoding, as only one entry of the input vector **x**_**t**_ is non-zero. As shown in Fig. 3a, this scalar product can be implemented with two memristors *w*_*t*+1_ and *w*_*t*_ from the critic network and one resistor *w*_fixed_, which are wired together in one row. In this manner, the first vector of the weight update can be mapped to the input voltages *U*_1_ = *α* × *r*(*s*_*t*_), *U*_2_ = *α* × *γ* and *U*_3_ = − *α* and the second vector to the weights *w*_fixed_ = 1, *w*_*t*+1_ = *V*(*s*_*t*+1_) and *w*_*t*_ = *V*(*s*_*t*_).

8 $$\Delta w({s}_{t})=\left(\begin{array}{c}\alpha \times r({s}_{t})\\ \alpha \times \gamma \\ -\alpha \end{array}\right)\cdot \left(\begin{array}{c}1\\ V({s}_{t+1})\\ V({s}_{t})\end{array}\right)=\left(\begin{array}{c}{U}_{1}\\ {U}_{2}\\ {U}_{3}\end{array}\right)\cdot \left(\begin{array}{c}{w}_{fixed}\\ {w}_{t+1}\\ {w}_{t}\end{array}\right)$$

Since the reward term *α* × *r*(*s*_*t*_) is a feedback signal from the environment of the navigation task, it is implemented by the applied voltage *U*_1_ and requires *w*_fixed_ to be equal to one. A resistor is, thus, chosen to represent this constant term, but it could also be implemented using another memristor with a fixed conductance. In practice, the memristor conductances need to be converted to normalized weights, which results in adjusted input voltages *U*_1_–*U*_3_. A detailed explanation of the mathematical derivation of these voltages is provided in Supplementary Note 2.

### Error correction mechanism

In navigation tasks, when moving between states in its environment, an agent strives to choose actions that maximize the amount of reward it collects. A difference between the actual (the immediate reward the agent receives) and expected (the predicted reward the agents anticipates if it follows its current strategy) reward leads to a non-zero TD error 3^*rd*^ (equation (2)), which updates the actor *θ*_*ij*_ and critic weights *w*_*j*_ according to equation (1). This iterative adjustment of the weights drives the agent’s learning process towards a near-optimal set of state values *V*(*s*_*t*_) and policy *π*(*i*∣*s*_*t*_).

Our actor–critic RL framework calculates the weight updates Δ*w*_des_ directly in hardware through a subnetwork of two critic memristors (*w*_*t*_ and *w*_*t*+1_) along with a fixed-value resistor (*w*_fixed_) (Fig. 3a). Two sources of errors are introduced during the actual update: an error *ϵ*_1_ because of the nonlinear dependence of the weight update on the number Δ*p* of applied voltage pulses and an error *ϵ*_2_ because of the inherent noise in the memristor updates. Neither *ϵ*_1_ nor *ϵ*_2_ are known during the hardware update and are, therefore, contained in the new weight after the update. However, as the new weights directly represent the value estimates of the current (*V*(*s*_*t*_)) and next (*V*(*s*_*t*+1_)) state through *w*_*t*_ and *w*_*t*+1_, respectively, both error terms are taken into account during the subsequent iteration and, therefore, compensated through the in-memory calculation of the next desired weight update (Fig. 3a(iv)). They are, thus, trained away by the algorithm^11^, leading to an error correction mechanism. Similar mechanisms are present in other online training algorithms on memristors. However, these implementations require an external computation of the weight update to account for these error terms (that is, the gradient of the loss function in backpropagation is computed in software), preventing full in-memory training. By contrast, in our approach, the weight updates are computed in hardware according to equation (3) and implemented by the scheme shown in Fig. 3a. A mathematical description of the error correction mechanism is provided in Supplementary Note 4.

The error correction mechanism can compensate for non-idealities such as update noise or conductance drift. As such, it can also adapt to potential device degradation that occurs over long timescales. If conductance values change over time, the TD error is no longer equal to zero, naturally triggering retraining and thereby mitigating other hardware non-idealities as well. However, this requires that devices remain reprogrammable after degradation, that is, no permanent device failure has occurred.

We also investigated the impact of read accuracy (Fig. 3b) during the hardware weight update calculation on convergence. Specifically, we analysed how variations in this accuracy affect the convergence in the Morris water maze task (Supplementary Fig. 6). The simulation results show that our measured read accuracy has a negligible impact on the convergence and performs similarly to the ideal case with perfect accuracy.

### Evaluation of the error correction mechanism

The error correction mechanism was tested by solving the T-maze navigation task illustrated in Fig. 4 with in-software-emulated memristors and by inspecting the resulting standard deviation of the critic weights *w*_*j*_ as a function of the episode number. Specifically, we compared the case in which the errors *ϵ*_1_ (resulting from linear updates on nonlinear potentiation/depression curves) and *ϵ*_2_ (update noise) were included in the in-memory weight update calculation of the next iteration (with feedback) to the case in which they were omitted (no feedback). In the case where *ϵ*_1_ and *ϵ*_2_ were not fed back, errors were accumulated over different iterations of the learning algorithm, resulting in a higher standard deviation of the learned weights and a larger spread in the weights. We conducted 1,000 distinct simulated runs and extracted the mean along with error bars representing two standard deviations.

### Implementation of the T-maze experiment

The algorithm used for the T-maze experiment is based on the equations of TD learning presented in the ‘Analogue memristor synapses as active components of actor–critic networks’ section. The TD error (equation (2)) adapts the actor and critic synapses based on the learning rules given in equation (1) with the adjustable learning parameters *α*, *γ* and *T*. The reward function *r*(*s*_*t*_) is equal to 1 for state 6 (where a reward is present) and 0 otherwise. In all the runs, we set the discount factor *γ* to 0.9. Moreover, the optimal parameters for the learning rate and softmax temperature are determined through a grid search of simulated runs using in-software-emulated memristors (Supplementary Note 5), which yields *α* = 0.2 and *T* = 0.3, respectively. For all the experiments, the reward was placed in the left corner of the T-maze (state 6). Although the task involved a static reward, our actor–critic framework is also capable of learning in dynamic environments in which the reward location changes slowly over time, either smoothly or abruptly. In such cases, the actor and critic weights would slowly adapt through updates driven by the TD error. The speed of relearning could be increased further by the use of a global signal conveying uncertainty or surprise^37,80,81^.

**Extended Data Fig. 6:**
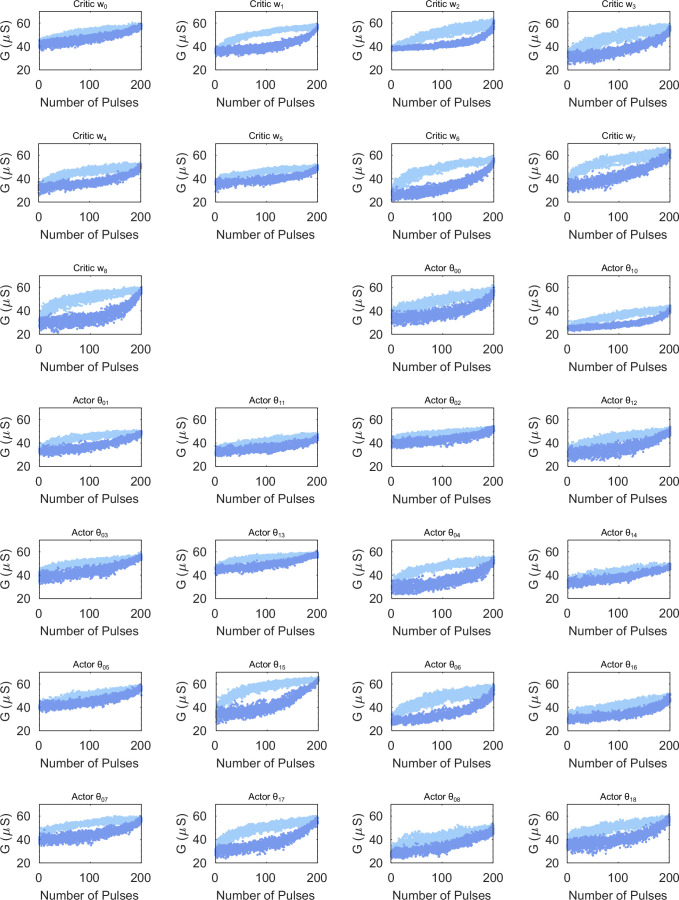
Measured memristor potentiation and depression curves used as a reference to parametrise the memristor behaviour emulated in software. These in-software-emulated memristors are used in the T-maze and Morris water maze navigation tasks (Figs. 4 and 5 of the main text). Each subplot consists of 10 cycles. The 9 critic weights are denoted as *w*_*j*_ and the 18 actor weights as *θ*_*ij*_.

As mentioned in the main text, the actor–critic network comprises 27 synaptic weights in total, including 9 × 2 = 18 actor weights (*θ*_*ij*_) and 9 × 1 = 9 critic weights (*w*_*j*_). Each of these weights is implemented by a different hardware memristor. In each run, two out of the nine critic weights are represented by physical devices and updated in hardware via online training. Due to experimental constraints (Supplementary Fig. 7), we are limited to operating and training only two memristors per run at the same time. The behaviour of the other critic and actor weights is, thus, emulated in software. Each of them relies on the fitted characteristics of a distinct memristor, including its potentiation/depression curves, cycle-to-cycle variability, nonlinearity and update noise (Extended Data Fig. 6 shows the measured potentiation and depression curves of all 27 in-software-emulated memristors). The same two hardware devices additionally implement the in-memory weight update calculation, as introduced in Fig. 3a.

Due to experimental constraints, including the availability of only four probe needles on the probe station and a limited number of output channels on the arbitrary waveform generators, we were restricted to operating and updating two memristors in parallel per run.

### Implementation of the Morris water maze experiment

As the simulated environment is continuous, Gaussian RBFs are used as the input layer of our actor–critic network (Fig. 1c). They create a representation of the current agent’s location in the maze, which is encoded by the activation **x**_**t**_ of 121 overlapping RBFs that are centred at evenly spaced grid points in an 11 × 11 layout. The components of **x**_**t**_ become larger as the agent moves closer to the corresponding grid point. The representation of positions through an RBF input layer enables to solve the complex water maze navigation task in continuous space by learning actions and state values in a single subsequent layer, thereby substantially reducing the required neural network size compared with multilayer networks^11,37,38^. Our choice of a fixed RBF grid with evenly spaced grid points is sufficient for the types of task analysed in this work, where the reward location is static and the environment obstacles placed uniformly across the space. However, if it is not known a priori where higher spatial resolution is needed, a more flexible place-cell representation would be advantageous. For example, self-organizing maps or similar unsupervised algorithms could be employed, as they typically rely on local learning rules^58,61,82^, and are, therefore, fully compatible with our in situ, local learning framework.

To navigate through the maze, the agent chooses among eight possible actions (Fig. 5a). Following the actor–critic network shown in Fig. 1c, each place cell is connected to one critic neuron and eight action neurons. In total, the actor–critic network comprises 1,089 synaptic weights, including 121 × 1 critic weights and 121 × 8 = 968 actor weights. The behaviour of all these weights is emulated in software, with all the weights initialized to zero, which showed the fastest convergence (Supplementary Fig. 8). We use the same 27 potentiation/depression measurements as in the T-maze (Extended Data Fig. 6) as the basis for the in-software-emulated memristors. Although the number of weight update curves is much smaller than the total number of synaptic weights, device-to-device variability is captured by randomly assigning these measured curves to the network weights. For each device, the emulated weight updates incorporate cycle-to-cycle variability, nonlinearity and update noise. Compared with the T-maze case in which distinct cycles were chosen at each iteration, here cycle-to-cycle variability and update noise are combined within a single error term $\sigma$. For each memristor, this parameter is extracted from overlapping all ten measurement potentiation/depression cycles (similar to Fig. 2f). By varying $\sigma$, we can properly investigate the effect of the total update error on our simulation runs. Moreover, we analysed the impact of actor weight initialization and granularity (that is, the number of pulses between minimum and maximum conductance) on the convergence speed (Supplementary Fig. 8).

### Extension to deep networks

In our navigation framework, a single RBF-based input layer is sufficient to encode a representation of the environment. This representation is rich enough for learning actions and state values in a single subsequent layer, making deep RL unnecessary^58,61,62^. A representation with approximate RBFs could be the result of a generic preprocessing pipeline, for example, with a deep convolutional neural network that serves as a foundation model and transforms arbitrary input images, or other sensor data, into high-level representations^61,83–85^. All weights of the preprocessing pipeline could be mapped onto memristors, with each layer implemented as a crossbar array. Only the last layer—the actor–critic one—would be trained in situ on a specific task, using our three-factor learning rule and in-memory weight update scheme.

One limitation of the proposed approach is the limited adaptability to new environments due to the fixed input layer(s). The application of three-factor learning rules with local plasticity to the case of self-supervised representation learning provides an alternative to extend our approach to deep neural networks^84,86,87^. These biologically inspired learning rules rely on layer-specific loss functions and eliminate the need for the backpropagation of error signals. To illustrate this, we have used the local three-factor rule, named CLAPP^84,88^, in simulation in a deep network comprising six layers. The deep network was pretrained on the STL10 database. We then kept the weights fixed and applied inputs from simulated views of a three-dimensional T-maze environment with images on the walls (Supplementary Fig. 9). The representation layer (layer 6 of the deep network) was rich enough that it could be used as input to our simulated (one-layer) actor–critic network, which learns the navigation task in fewer than 20 trials. However, these rules are currently an active field of research and it is too early to attempt an implementation memristor-based architectures.

### Comparison with other RL algorithms and local learning rules based on backpropagation approximations

The actor–critic TD learning algorithm lends itself particularly well to an in-memory implementation compared with the most-common RL algorithms such as Q-learning, SARSA or Monte Carlo methods^11^. Whereas Monte Carlo methods are not compatible with online learning^11^, Q-learning is an online, although off-policy method, which prevents efficient in-memory weight updates. SARSA theoretically allows for a similar hardware implementation as TD learning with actor–critic networks, but the latter directly learns and updates the action policy over time, a feature that makes it both resistant to function approximation errors and better suited to complex environments^11^. Since our bio-inspired algorithm employs RBFs to represent the agent’s location, a single subsequent layer combined with a local learning rule is sufficient to learn both actions and state values, realizing complex navigation tasks^38,58,61^. Owing to the local learning rule, only individual weight updates on a small subset of all memristor devices are performed.

Within our developed framework, hardware memristors are not only employed as synaptic weights for online learning but also for the calculation of weight updates. Compared with existing in-memory weight update calculations, where updates are solely based on the sign of the weight change and thus imprecise^31,32^, our method computes exact weight updates. When updating the memristors, no additional error mitigation schemes such as write–verify algorithms are necessary as opposed to other weight update schemes^89,90^, thereby simplifying the control circuitry^27,31,91^. Hence, the proposed approach minimizes off-device computations and avoids weight read-outs so that the main task of the software reduces to environment interactions.

**Extended Data Fig. 7:**
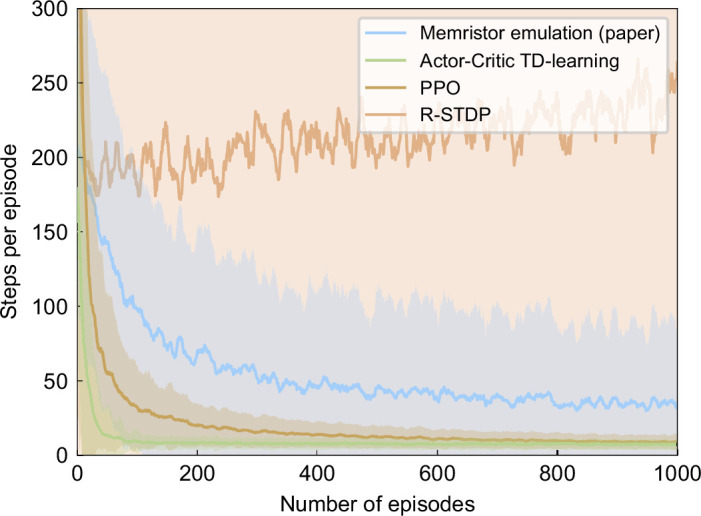
Actor–critic TD learning (floating-point and memristor emulation) compared to PPO and rate-based R-STDP. The performance of the actor-critic TD learning algorithm is comparable to PPO in the floating-point software implementation, whereas convergence slows down for the memristor emulation due to update noise (Extended Data Fig. 4). R-STDP does not converge, as updates effectively only happen when the reward is reached and not when a non-zero TD error is present. The memristor model represents the mean of 100 independent simulation runs, while the PPO results are averaged over five random seeds. A moving average over 10 episodes was applied to improve comparability between runs.

Our methodology contrasts with modern deep RL methods such as deep Q-networks and proximal policy operation (PPO) that rely on error backpropagation across multiple layers^11^ and are, therefore, less biologically plausible^18^ than our actor–critic TD learning approach, where both actions and state values are learned using a single layer. We note that deep RL methods^14^ train all layers on a given task or set of tasks^13^. However, in our approach, we assume that a good representation of the environment can be achieved independently of the task, using, as preprocessing, a foundation model trained with modern self-supervised learning algorithms^92–95^ on large datasets. In line with existing foundation models, we expect that a representation built by the foundation model is useful for many different tasks. Most importantly, although deep RL algorithms have demonstrated strong performance in many deep RL tasks^13^, they go beyond what is needed to solve navigation tasks^38,58,61^. In Extended Data Fig. 7, we directly compare our actor–critic TD learning algorithm with PPO and R-STDP implementations on the Morris water maze task, using the same RBF input representation and an identical network structure consisting of a single layer. While the software implementations of actor–critic TD learning and PPO achieve similar performance, the memristor emulation performs slightly worse due to the presence of non-idealities in the weight updates, and R-STDP does not converge at all. Unlike TD learning, where weight updates happen whenever a non-zero TD error (a reward prediction error) is present, updates in R-STDP only take place when the reward is reached.

As an alternative to directly implementing local three-factor learning rules, yet avoiding the biological limitations of backpropagation, several approximations of the backpropagation algorithm have emerged in recent years^18^. A notable example is the proposed memristor-based architecture employing direct feedback alignment^96^. Although these methods are compelling, it is important to highlight a key distinction: in our framework, the TD error acts as a scalar, one-dimensional global error signal, in contrast to the high-dimensional error signals used in both backpropagation and direct feedback alignment. This scalar error enables fully local learning by eliminating the need for network-wide error propagation (as required in direct feedback alignment) and allows the same modulatory signal to be broadcast uniformly to all synapses, unlike the synapse-specific feedback used in approaches such as that in ref. ^96^.

### Energy consumption and latency estimation of a crossbar-level implementation

The energy consumption and latency of the actor–critic TD learning algorithm was calculated, focusing specifically on the operations that can be performed in hardware to highlight the potential of a crossbar implementation of our framework (Extended Data Table 1). We compared three different cases: ‘this work’, ‘hybrid’ and ‘software’, where ‘hybrid’ refers to other works that employ memristors within the RL algorithm and ‘software’ to an implementation without memristors. Each algorithmic operation performed in ‘software’ is assumed to be executed on an NVIDIA A100 40-GB GPU. The operations performed in ‘hardware’ are assumed to be implemented on a crossbar array, namely, the one proposed in Supplementary Note 7. For both GPU and memristor operations, we consider a ‘standard’ case and an ‘optimal’ case, as well as a ‘compute’ scenario specifically for the GPU. The GPU is ideally fully utilized (‘optimal’ case), which results in the lowest latency and energy consumption, whereas ‘standard’ is a more realistic utilization scenario, such as that in ref. ^97^. ‘Compute’ provides a reference for the energy consumed solely by computation, excluding any overhead from fetching or storing weights in memory. As basis for the energy and latency calculations, we employ the values presented in Table 2 and Supplementary Table S1 of ref. ^97^. For the memristor implementations, we consider a standard case using the pulse widths employed in this work, as well as an optimal case with a 60-ns pulse width for all the operations, similar to what has been demonstrated in the past for the same HfO_2_–CMO cells^98^ (Supplementary Note 8 provides more details on the effect of the pulse width on energy consumption). Furthermore, we assume all memristors to be in the low-resistance state of 50 μS, and that each weight update consists of three reset pulses of 10 μs (standard case) or four set pulses of 60 ns (optimal case), each representing the worst-case scenario in terms of energy consumption during the water maze emulation. For all vector–matrix and vector–vector calculations, we consider the same mapping that we used in the hardware calculation of Δ*w* in the T-maze, which results in a maximum voltage of 0.1 V applied to a memristor. Here we assume that 0.1 V is applied to all memristors during the vector–matrix and vector–vector calculations.

In the analysis of energy consumption and latency, we did not include the contribution of the peripheral circuitry. Analogue-to-digital digital-to-analogue converters are typically the main contributors to the energy consumption of memristor-based systems^99^. To minimize their negative impact, our framework avoids converting data between the digital and analogue domains by computing as many components of the algorithm as possible in memory. This is expected to further reduce the energy consumption and latency compared with other memristor-based systems.

## Supplementary information

Supplementary Information Supplementary Figs. 1–17, Notes 1–8, Table 1 and references.

## Figures and Tables

**Extended Data Table 1:**
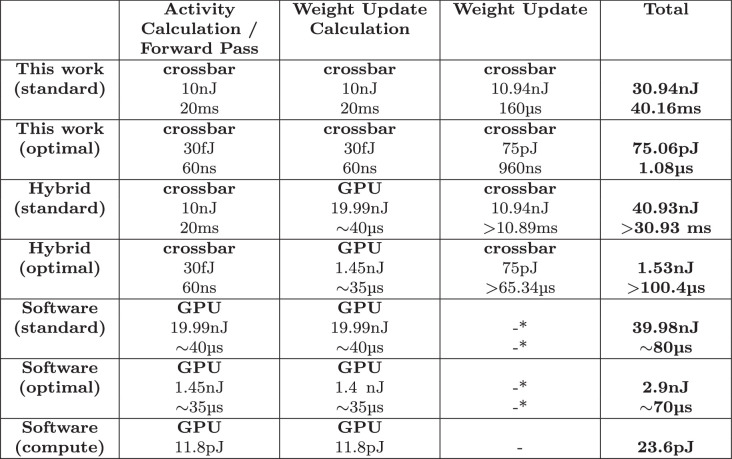
Energy consumption and latency of the actor-critic TD learning algorithm for three different scenarios (i) a crossbar implementation of our framework (‘This work’), (ii) common approaches of using memristors within RL applications (‘Hybrid’) and (iii), a full software implementation executed on a GPU

The energy consumption is given per single operation and is normalized to the number of weights. Here, we assume an fp16 precision of the GPU. For both the activity and weight update calculation on the GPU we assumed a vector-matrix operation. In practice, the weight update calculation would be a vector-vector multiplication which cannot benefit from the same degree of parallelism and would be less efficient. The latency values for the operations performed on the GPU are taken from^97^, and were extracted for the specific matrix sizes in their algorithm. Thereby they can only serve as a rough reference. ⋆: The energy consumption and latency for the GPU operation of vector-matrix multiplications already includes fetching the data from memory and storing the results.

## Data Availability

The figure files used in this study are available via Zenodo at 10.5281/zenodo.15740718 (ref. ^100^), whereas the datasets for the T-maze and Morris water maze experiments are available via GitHub at https://github.com/ztill/TD_learning_on_memristors/.

## Code availability

The source code for the TD learning framework is available via Zenodo at 10.5281/zenodo.17315889 (ref. ^101^) and via GitHub at https://github.com/ztill/TD_learning_on_memristors/.
