# Supplementary material for: Actor–critic networks with analogue memristors mimicking reward-based learning
Source: Nat Mach Intell. 2025 Dec 9;7(12):1939–53. doi: 10.1038/s42256-025-01149-w (PMC12719229; doi:10.1038/s42256-025-01149-w)
Supplement: Supplementary file 1 — Supplementary Figs. 1–17, Notes 1–8, Table 1 and references. [file 42256_2025_1149_MOESM1_ESM.pdf]

nature machine intelligence

Supplementary information https://doi.org/10.1038/s42256-025-01149-w

Actor–critic networks with analogue memristors mimicking reward-based learning

In the format provided by the authors and unedited

Contents

Supplementary Figures **S2**

Supplementary Figure 1: Actor-critic architectures in biology and reinforcement learning . . . S2

Supplementary Figure 2: Forming step of the measured memristors S3

Supplementary Figure 3: Experimental setups used for the DC, dynamic and T-maze navigation measurements S5

Supplementary Figure 4: Impact of the softmax temperature T on exploration and exploitation in the T-maze navigation task S5

Supplementary Figure 5: Comparison of the employed memristor model with full non-linearity, linear memristors, and ideal floating-point training S6

Supplementary Figure 6: Simulations showing the impact of read accuracy S7

Supplementary Figure 7: Pictures of the experimental setup used for the T-maze navigation task S8

Supplementary Figure 8: Effect of actor weight initialization and granularity S9

Supplementary Figure 9: Actor-critic learning in the T-maze using CLAPP S12

Supplementary Table 1: Comparison of reinforcement learning demonstrations on memristive hardware S12

Supplementary Notes **S12**

Supplementary Note 1: Simplified formulas for the case of one-hot encoding **S12**

Supplementary Note 2: Weight normalization in the in-memory weight update calculation **S13**

Supplementary Note 3: In-software-emulated memristors **S15**

Supplementary Note 4: Error correcting mechanism **S21**

Supplementary Note 5: Grid search heatmaps for memristors **S22**

Supplementary Note 6: T-maze navigation task using hardware actor weights **S27**

Supplementary Note 7: TD learning framework on crossbar arrays **S29**

Supplementary Note 8: Energy consumption during the in-memory weight training **S33**

References **S35**

Supplementary Figures

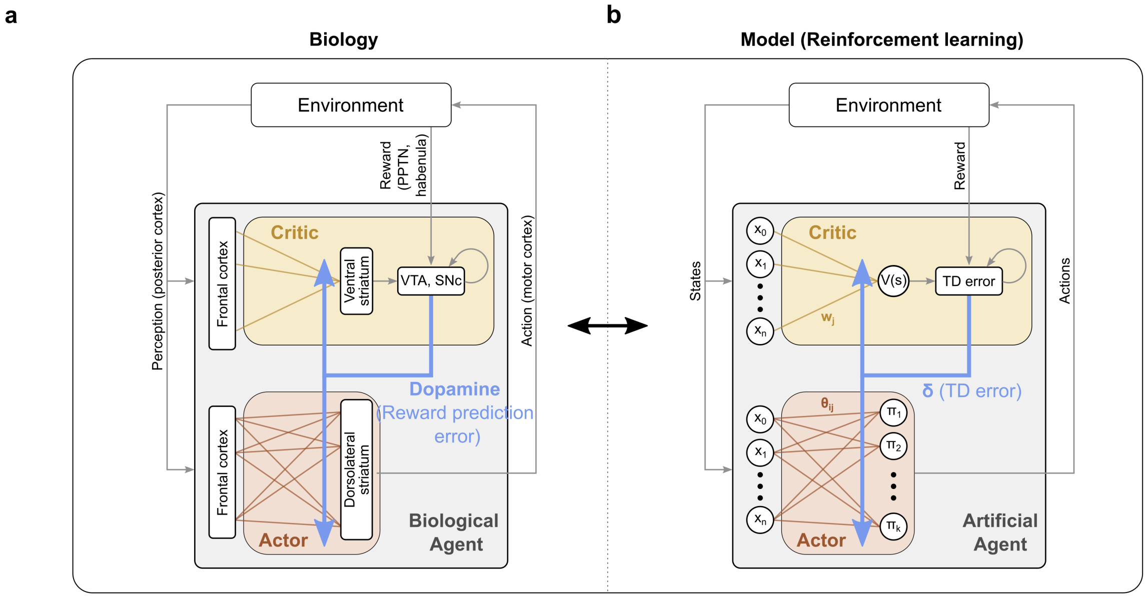

Supplementary Figure 1| Actor-critic architectures in (a) biology and (b) reinforcement learning. The figure was recreated and adapted from [1].

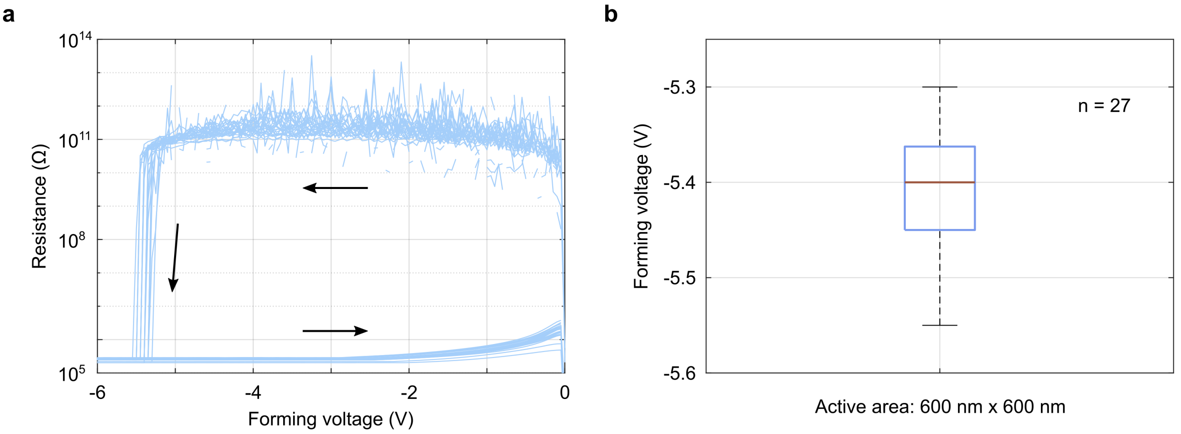

Supplementary Figure 2| Forming step of the measured memristors. (a) Forming step applied to the 27 devices employed in the T-maze navigation task presented in Fig. 4 of the main text. These memristors feature an active area of 600 nm × 600 nm and were formed by applying a negative voltage to the top electrode. To prevent permanent dielectric breakdown [2], a current compliance (CC) of I_{CC}=100 µA was utilized in conjunction with a 10 kΩ resistor placed in series. Note that the presented values include the 10 kΩ from the resistor. **(b)** Boxplot displaying the forming voltages extracted from the 27 devices (n = 27). The center line indicates the median forming voltage (-5.4 V), with the first and third quartiles measuring -5.45 V and -5.36 V, respectively. The whiskers extend to the minimum (-5.55 V) and maximum (-5.3 V) values. This distribution underscores the uniform and consistent forming behaviour observed across the memristors.

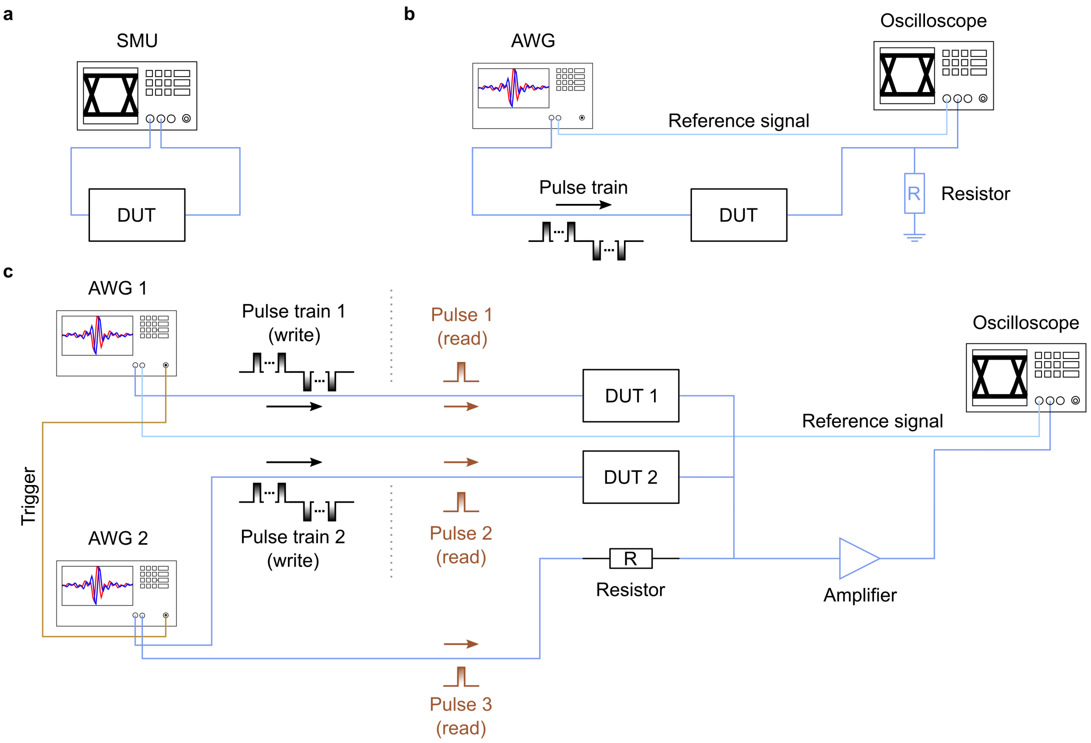

Supplementary Figure 3| Experimental setups used for the DC, dynamic and T-maze navigation measurements. **(a)** Experimental setup used for the DC characterization (Fig. 2d of the main text). It comprises a source measure unit (SMU) to read out the resistance states. An external series resistor was used during forming to impose a current compliance (CC), while no external resistor was utilized for resistance-voltage (R-V) measurements. In the latter, the current passing through the device under test (DUT) was self-limited by the active layers (Conductive Metal Oxide (CMO) and HfO_2) and the 13 nm thin TiN layer, acting as series resistors. **(b)** Experimental setup used for the dynamic characterization. The electrical pulse trains for the potentiation and depression curves (Fig. 2e of the main text) are created by an arbitrary waveform generator (AWG). As the AWG is not capable of imposing a compliance current, a 10 $k\Omega$ resistor is connected to the memristors to limit the current passing through them. The layers of the device itself also exhibit current-limiting capabilities as mentioned above. The weights (conductance states) of the potentiation and depression curves are determined via the voltage drop across the 10 $k\Omega$ resistor using an oscilloscope. **(c)** Experimental setup used for the T-maze navigation task (Fig. 4 of the main text). For the weight update a pulse train is applied to the specific memristor that needs to be updated. To read the conductance of a memristor (only as reference and not within the learning algorithm) a read pulse is applied and the current is measured using a transimpedance amplifier and an oscilloscope. For the hardware weight update calculation three voltage signals are applied simultaneously. The resulting current is amplified by the transimpedance amplifier and corresponds to the weight update Δw ("In-memory weight update calculation", box in Fig. 3a of the main text) . It is recorded using the oscilloscope. Since each AWG has two output channels, two distinct waveform generators are used. A trigger signal between both AWGs enables synchronization. The current measurement setup introduces a significant latency due to the communication bottleneck between the oscilloscope and AWG on the one hand and the control PC on the other hand. A low-latency implementation of the complete system would require an integrated solution combining an FPGA or ASIC, ADCs, DACs, and the memristor chip on a single circuit board.

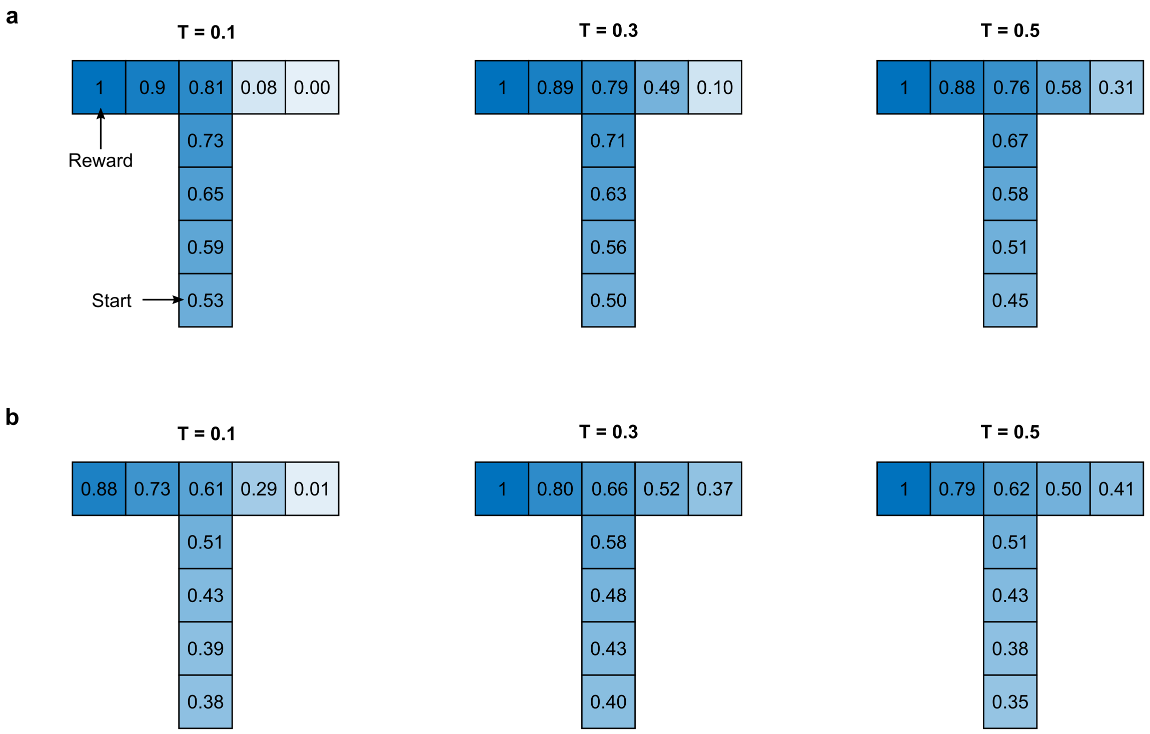

Supplementary Figure 4| Impact of the softmax temperature T on exploration and exploitation in the T-maze navigation task. The effect of three different softmax temperature factors (0.1, 0.3, 0.5) is shown by means of the T-maze navigation task value maps. These displayed values represent the trained critic weights after 200 episodes. They were extracted from simulations and are the means of 100 distinct runs. It can be seen that states in the right corner are explored more often when the temperature factor is high. **(a)** Value maps with learned critic weights based on ideal (perfectly linear) conductance updates. **(b)** Same as **(a)**, but for devices incorporating update noise and cycle-to-cycle variability.

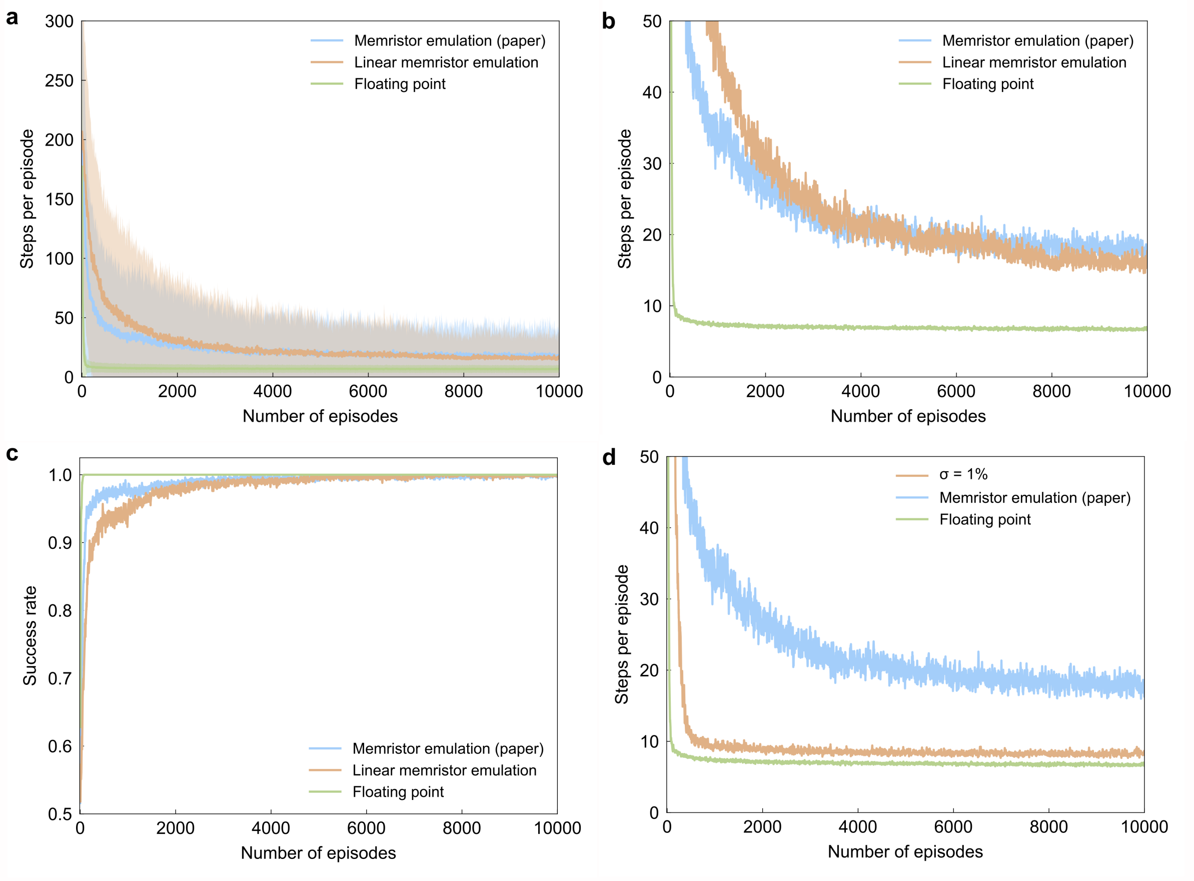

Supplementary Figure 5| Comparison of the employed memristor model with full non-linearity, linear memristors, and ideal floating-point training (a-c), and effect of reduced device noise on learning performance (d). Each curve represents the average of 100 independent simulation runs, with an applied running average of 10 episodes to improve comparability between the runs.

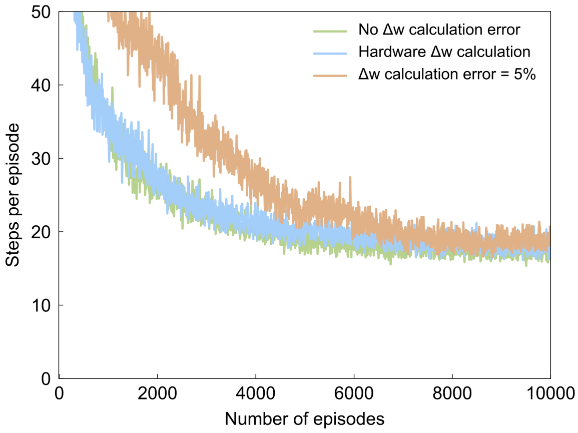

Supplementary Figure 6| Simulations showing the impact of read accuracy during the hardware weight update calculation on convergence in the Morris water maze navigation task. Three cases are compared: (i) ideal hardware calculation with no error, (ii) actual measured error of the hardware calculation (mean error of 0.0061 and standard deviation of 0.0096, i.e., 1.0% of the [0, 1] weight range), and (iii) a high-error case (standard deviation of 0.05, i.e., 5% of the [0, 1] weight range). While the measured read accuracy has a negligible effect on convergence and performs similarly to the ideal case with perfect read accuracy, a significantly higher error of 5% leads to slower convergence. Each curve represents the average of 100 independent simulation runs, with an applied running average of 10 episodes to improve comparability between the runs.

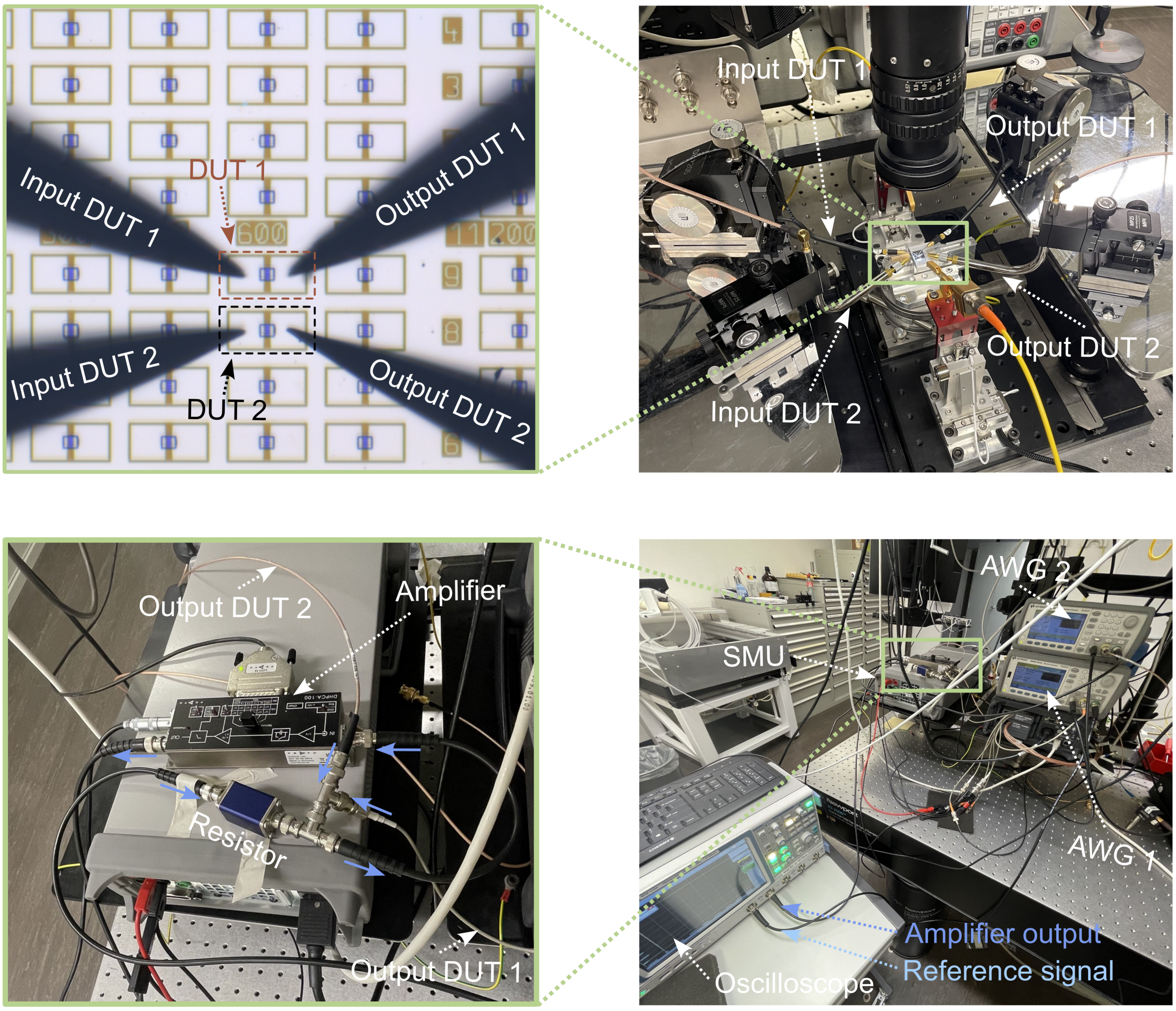

Supplementary Figure 7| Pictures of the experimental setup used for the T-maze navigation task.

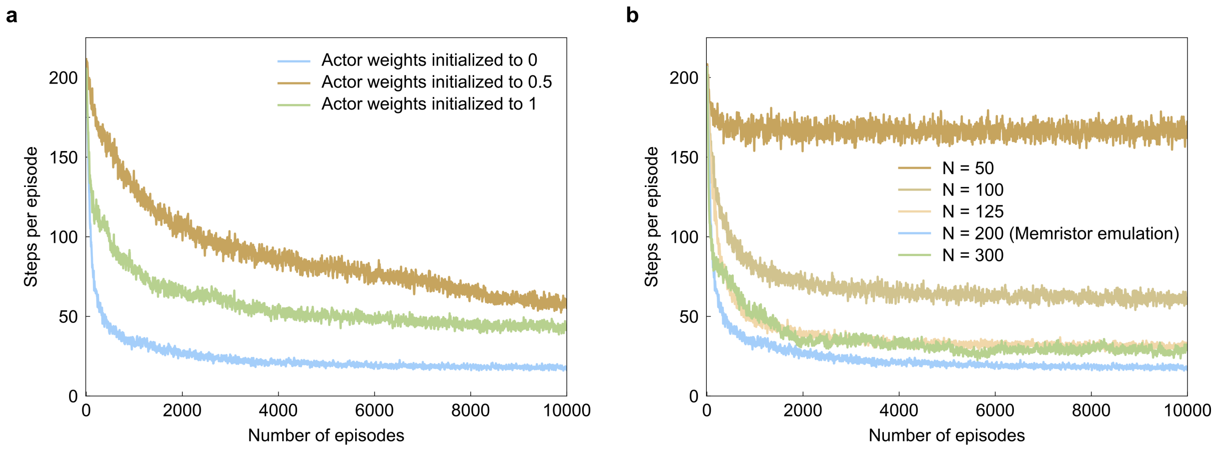

Supplementary Figure 8| Effect of (a) actor weight initialization and (b) granularity (number of pulses between minimum and maximum conductance) on the convergence rate in the Morris water maze navigation task. Each curve represents the average of 100 independent simulation runs, with an applied running average of 10 episodes to to improve comparability between the runs.

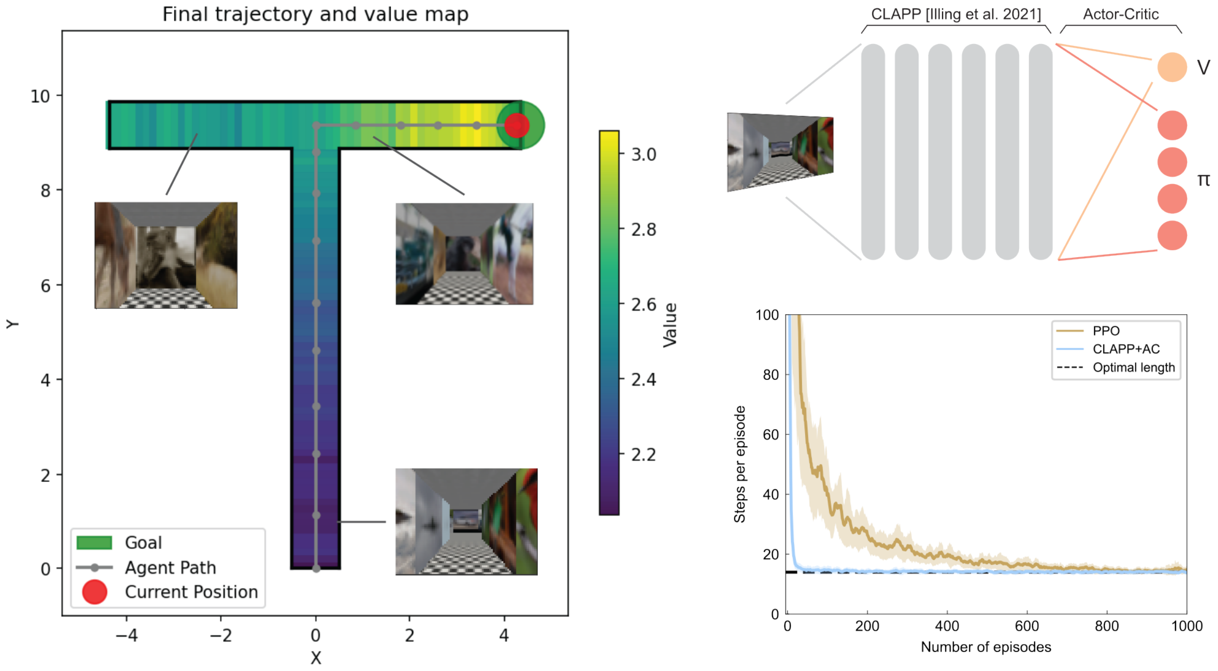

Supplementary Figure 9| Our actor-critic (AC) algorithm can learn optimal policies by leveraging representations extracted by a deep network trained with local three-factor learning rules [3]: (**Left**) Top-view of a T-maze simulated in a 3D environment: pictures show input images given to the agent in different points of the maze. To orient the agent along the maze, images from the STL-10 dataset are pasted on the walls. The agent can take four possible actions: move north, south, east, or west. To avoid memorization of only few specific input views, the agent's step length is stochastic, with an average length of 1 and standard deviation of 0.2. In grey, we see a successful agent's trajectory, each dot represents the landing position after every action. The T-maze is color-coded based on the values (V) learned by the critic neuron, we see that the algorithm successfully learns to assign higher expected rewards closer to the goal. (**Top right**) A 6 layer CNN trained with local plasticity rules (CLAPP [3]) on the STL-10 dataset is used to process input image views of the T-maze. Our single layer actor-critic algorithm receives as input the representations extracted from the CNN's 6th layer. The CNN weights are frozen while the actor-critic learns optimal critic and policy weights. (**Bottom right**) In less than 20 episodes the algorithm learns the optimal trajectory to the reward, demonstrating that, with good representations extracted from pixels, a single layer actor-critic is sufficient to learn navigation tasks. In contrast, a PPO implementation with no pretraining requires around 800 episodes to converge when starting from pixels, as the input representations must first be learned through a convolutional neural network, resulting in a slower learning. The code to reproduce these results is available in the associated GitHub repository.

Supplementary Table 1 |Comparison of reinforcement learning demonstrations on memristive hardware.

	This work	Alam et al. [4]	Bianchi et al. [5]	Dai et al. [6]	Dalgaty et al. [7]	Lin et al. [8]	Lu et al. [9]	Wang et al. [10]
**Memristor technology**	TiN/CMO/ HfO_2/TiN (VCM)	N.A.	TiN/TiO_x/ Ti/TiN (VCM)	Ti/TiO_x/Pt (VCM)	TiN/HfO_2/ Ti (VCM)	TiN/TaO_x/ HfO_x/TiN (VCM)	C-doped $Ge_2Sb_2Te_5$ (PCM)	Pd/HfO_2/Ta (VCM)
**Architecture**	Actor-Critic Network	Deep Q-Network	SNN-like	Q-Network	Memristor Bayesian DNN	Memristor Bayesian DNN	Q-Network	Deep Q-Network
**RL algorithm**	TD Learning	Q-Learning (Backpropagation)	”Neuromorphic Q-Learning”	SARSA	Memristor-based MCMC	Proximal Policy Optimization (Backpropagation)	SARSA	Q-learning (Backpropagation)
**Bio-plausibility**	high	low	high	moderate	low	low	moderate	low
**Online learning**	yes	yes	yes	yes	yes	no	yes	no
**Readouts of weights**	no	yes	yes	yes	no	yes	yes	yes
**In-memory action computation**	yes	no	yes	no	yes	no	no	no
**In-memory weight update calculation**	yes	no	no	no	no	no	no	no

Supplementary Notes

Supplementary Note 1: Simplified formulas for the case of one-hot encoding

In the general case, each state is encoded by an input activation vector $\mathbf{x_t}$ with multiple non-zero values. However, for tasks with discretized states such as the T-maze considered in the main text, the input vector is one-hot encoded, meaning one entry of the vector is one and all others are zero, e.g., $s_t = 3 \rightarrow \mathbf{x_t} = [0, 0, 1, 0, 0, 0, 0, 0, 0]$. This assumption simplifies the formulation of the actor-critic TD learning problem. Most importantly, each critic weight w_j corresponds to exactly one state which leads to a simplified value function:

$$V(s_t) = \mathbf{w}^T \mathbf{x_t} = w_t \tag{S1}$$

This in turn results in a simplified TD error formula:

$$3^{rd} = \delta_t = r(s_t) + \gamma \times w_{t+1} - w_t \tag{S2}$$

As the Hebbian term of the critic is equal to the one-hot encoded $\mathbf{x_t}$, the critic update rule reduces to a simpler expression:

$$\Delta w(s_t) = \alpha \times 3^{rd} \tag{S3}$$

For the actor network, the formulas for $\mathbf{h} = \theta^T \mathbf{x_t}$ as well as $H^{act}(i, j)$ and consequently the update rule remain the same. However, as there is only one input neuron that is active (j) and connected via synaptic weights to two actions, only these two weights are updated.

Supplementary Note 2: Weight normalization in the in-memory weight update calculation

The in-memory weight update calculation for the critic weights in the T-maze task can be written as a scalar product of two vectors, as introduced in Eqs. (4) and (5) of the Methods section. The value estimates $V(s_{t+1})$ and $V(s_t)$ are represented by the weights w_{t+1} and w_t, corresponding to normalized conductance values. Because the conductance states of the memristors (G_{fixed}, G_{t+1}, G_t) are not intrinsically normalized, the input voltages U_1 to U_3 must be scaled to account for this difference. Concretely, we want to find scaled input voltages so that:

$$\Delta w(s_t) = \begin{pmatrix} U_1 \\ U_2 \\ U_3 \end{pmatrix} \cdot \begin{pmatrix} w_{fixed} \\ w_{t+1} \\ w_t \end{pmatrix} = \begin{pmatrix} U_{1,scaled} \\ U_{2,scaled} \\ U_{3,scaled} \end{pmatrix} \cdot \begin{pmatrix} G_{fixed} \\ G_{t+1} \\ G_t \end{pmatrix} \tag{S4}$$

where the input voltages are given by $U_1 = \alpha \times r(s_t)$, $U_2 = \alpha \times \gamma$ and $U_3 = -\alpha$. As mentioned in the main text, the term w_{fixed} is constant and can be implemented with a resistor G_{fixed}. As $w_{fixed} = 1$ (Eq. (5) in the Methods section), Eq. (S4) can be written as:

$$\Delta w(s_t) = \begin{pmatrix} U_1 \\ U_2 \\ U_3 \end{pmatrix} \cdot \begin{pmatrix} 1 \\ w_{t+1} \\ w_t \end{pmatrix} = \begin{pmatrix} \frac{U_1}{G_{fixed}} \\ U_2 \\ U_3 \end{pmatrix} \cdot \begin{pmatrix} G_{fixed} \\ w_{t+1} \\ w_t \end{pmatrix} \tag{S5}$$

Next, we define our conductance normalization condition:

$$w = \frac{G - G_{min}}{G_{max} - G_{min}} \tag{S6}$$

where G is the measured conductance state of G_{t+1} and G_t, while G_{min} (G_{max}) corresponds to the minimum (maximum) conductance of the memristor potentiation/depression characterization curve. Replacing the normalized conductance values w_{t+1} and w_t by their expression in Eq. (S6) yields:

$$\Delta w(s_t) = \begin{pmatrix} \frac{U_1}{G_{fixed}} \\ U_2 \\ U_3 \end{pmatrix} \cdot \begin{pmatrix} G_{fixed} \\ \frac{G_{t+1} - G_{min,t+1}}{G_{max,t+1} - G_{min,t+1}} \\ \frac{G_t - G_{min,t}}{G_{max,t} - G_{min,t}} \end{pmatrix} = \begin{pmatrix} \frac{U_1}{G_{fixed}} \\ \frac{U_2}{G_{max,t+1} - G_{min,t+1}} \\ \frac{U_3}{G_{max,t} - G_{min,t}} \end{pmatrix} \cdot \begin{pmatrix} G_{fixed} \\ G_{t+1} - G_{min,t+1} \\ G_t - G_{min,t} \end{pmatrix} \tag{S7}$$

Which can be re-written as follows:

$$\Delta w(s_t) = \begin{pmatrix} \frac{U_1}{G_{fixed}} \\ \frac{U_2}{G_{max,t+1} - G_{min,t+1}} \\ \frac{U_3}{G_{max,t} - G_{min,t}} \end{pmatrix} \cdot \begin{pmatrix} G_{fixed} \\ G_{t+1} \\ G_t \end{pmatrix} - \frac{U_2 \times G_{min,t+1}}{G_{max,t+1} - G_{min,t+1}} - \frac{U_3 \times G_{min,t}}{G_{max,t} - G_{min,t}} \tag{S8}$$

To obtain a scalar product, we introduce the terms $A = -\frac{U_2 \times G_{min,t+1}}{G_{max,t+1} - G_{min,t+1}}$ and $B = -\frac{U_3 \times G_{min,t}}{G_{max,t} - G_{min,t}}$ and add them to the first vector element:

$$\Delta w(s_t) = \begin{pmatrix} \frac{U_1 + A + B}{G_{fixed}} \\ \frac{U_2}{G_{max,t+1} - G_{min,t+1}} \\ \frac{U_3}{G_{max,t} - G_{min,t}} \end{pmatrix} \cdot \begin{pmatrix} G_{fixed} \\ G_{t+1} \\ G_t \end{pmatrix} \tag{S9}$$

Correspondingly, we can now define three scaled input voltages $U_{1,scaled} = \frac{U_1 + A + B}{G_{fixed}}$, $U_{2,scaled} = \frac{U_2}{G_{max,t+1} - G_{min,t+1}}$ and $U_{3,scaled} = \frac{U_3}{G_{max,t} - G_{min,t}}$ and end up with:

$$\Delta w(s_t) = \begin{pmatrix} U_{1,scaled} \\ U_{2,scaled} \\ U_{3,scaled} \end{pmatrix} \cdot \begin{pmatrix} G_{fixed} \\ G_{t+1} \\ G_t \end{pmatrix} \tag{S10}$$

which is equal to the right-hand side of Eq. (S4).

In summary, the weight update of the in-memory weight update calculation can be realised with non-normalized conductance values by applying the following scaled input voltages:

$$U_{1,scaled} = \frac{1}{G_{fixed}} \times \left(\alpha \times r(s_t) - \frac{\alpha \times \gamma \times G_{min,t+1}}{G_{max,t+1} - G_{min,t+1}} + \frac{\alpha \times G_{min,t}}{G_{max,t} - G_{min,t}} \right) \tag{S11}$$

$$U_{2,scaled} = \frac{\alpha \times \gamma}{G_{max,t+1} - G_{min,t+1}} \tag{S12}$$

$$U_{3,scaled} = \frac{-\alpha}{G_{max,t} - G_{min,t}} \tag{S13}$$

Importantly, all these values remain constant throughout the learning task. They should be determined at the beginning of the process. In case that the conductance range is similar for all critic memristors, i.e., $G_{min} = G_{min,t+1} = G_{min,t}$ and $G_{max} = G_{max,t+1} = G_{max,t}$, the expression for the scaled voltages can be simplified and become independent of the memristor position in the network:

$$U_{1,scaled} = \frac{1}{G_{fixed}} \times \left(\alpha \times r(s_t) + \frac{\alpha \times (1 - \gamma) \times G_{min}}{G_{max} - G_{min}} \right) \tag{S14}$$

$$U_{2,scaled} = \frac{\alpha \times \gamma}{G_{max} - G_{min}} \tag{S15}$$

$$U_{3,scaled} = \frac{-\alpha}{G_{max} - G_{min}} \tag{S16}$$

Note that the only variable in these equations is the reward $r(s_t)$ which is given by the environment. All other terms are constant and do not require any computation prior to the weight update calculation.

Supplementary Note 3: In-software-emulated memristors

This section introduces the in-memory learning loop for the in-software-emulated memristors and discusses how the emulation of the measured analogue memristors is done in software.

In-memory loop for in-software-emulated memristors

The learning loop for in-software-emulated memristors is depicted in Supplementary Fig. 10. Compared to the hardware case, the weight update of in-software-emulated memristors is carried out using fitted models for each cycle that are extracted from measured potentiation and depression curves. During an update a random cycle is chosen, which mimics cycle-to-cycle variability [11], and a noise level is drawn from the fitted normal distribution of the update noise. This leads to an error ϵ_2 in software. On the other hand, the error term ϵ_1 is introduced by performing linear updates on non-linear model curves similar to the case of physical memristors.

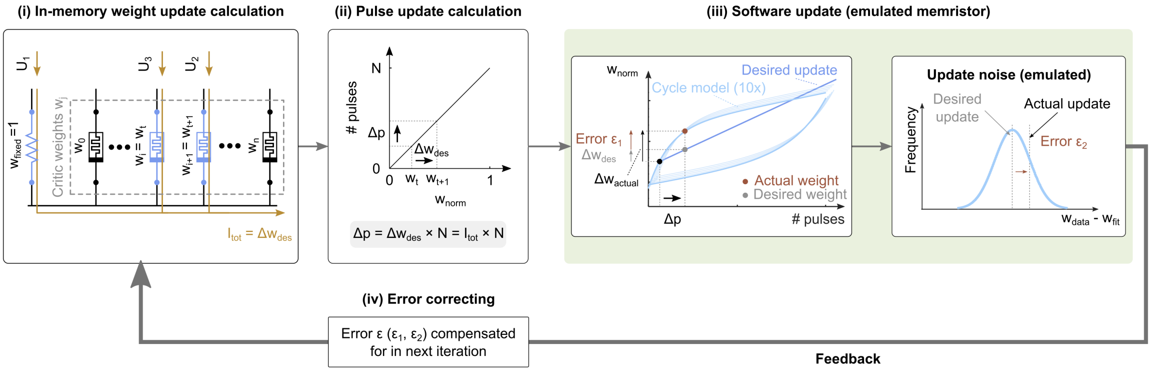

Supplementary Figure 10| In-memory learning loop for in-software emulated memristors The learning procedure is analogous to that of the hardware memristors (Fig. 3a of the main text), except that the in-memory weight update calculation (i) and the actual weight update (iii) are performed using in-software-emulated memristors.

Model and noise fitting

The emulation of the measured analogue memristors in software includes the fitting of each measured cycle with an analytical model that represents the reference weight update curve. Besides that, the update noise is quantified and translated into another fitting model.

First, the potentiation and depression curves of 10 cycles were measured for 27 different physical memristors (Extended Data Fig. 6). Identical pulse trains consisting of 200 set pulses at 2.5 V with a 1.5 μs pulse width for potentiation and 200 reset pulses at -2.7 V with a 10 μs pulse width for depression were used for these measurements, similar to Fig. 2e of the main text. Mean minimum G_{min} and maximum

G_{max} conductance values were extracted for each device based on the 10 cycles that were measured:

$$G_{min} = \frac{1}{10}\sum_{i=1}^{10} G_{set,i}(p = 0) \tag{S17}$$

$$G_{max} = \frac{1}{10}\sum_{i=1}^{10} G_{set,i}(p = N) \tag{S18}$$

where $G_{set,i}$ is the conductance value of the potentiation curve of cycle i, p is the pulse index, and N denotes the maximum number of applied pulses. The latter is equal to 200 in our case.

The potentiation and depression curve of each cycle i were then normalized using the calculated G_{min} and G_{max} values according to:

$$w_{set,i} = \frac{G_{set,i}}{G_{max} - G_{min}} \tag{S19}$$

$$w_{reset,i} = \frac{G_{reset,i}}{G_{max} - G_{min}} \tag{S20}$$

For each memristor, all 10 cycles were normalized by the same G_{min} and G_{max}.

Each normalized cycle was then fitted by an analytical model including the four fitting parameters, $\alpha, \beta, \gamma, \zeta$, similar to [11, 12]. The potentiation and depression curves are approximated as follows:

$$w_{set,fit}(p) = \alpha_{set} + \beta_{set} \times p - \gamma_{set} \times \exp\left(-\zeta_{set} \times p\right) \tag{S21}$$

$$w_{reset,fit}(p) = \alpha_{reset} + \beta_{reset} \times p + \gamma_{reset} \times \exp\left(\zeta_{reset} \times p\right) \tag{S22}$$

where p denotes the number of pulses. The model consists of a constant offset, a linear and an exponential term. It thereby captures both the non-linear and linear dependence of the data on the number of pulse p as well as the variance of G_{min} and G_{max} from one cycle to the other. Supplementary Fig. 11a demonstrates the accuracy of this fitting for a representative cycle out of the measured devices. The fits of the various cycles are the central components of the actor-critic reinforcement learning (RL) tasks as they serve as weight update curves for the in-software-emulated memristors. As long as the temporal difference (TD) error is non-zero, the number of pulses Δp leading to the weight update Δw_{ideal} in the ideal case is applied. The actual, non-ideal Δw_{act} is calculated with Eqs. (S21) and (S22) before adjusting the weight of the corresponding software memristor accordingly. As shown in Fig. 3a of the main text, we thereby assume a linear relation between Δp and Δw, which introduces the error term ϵ_1.

After performing this weight update, we include the influence of noise (ϵ_2) by adding Δw_{noise}, which is assumed to obey a normal distribution with all parameters extracted from experimental data. More precisely, to model this update noise for each cycle i, we first extract the deviation of the normalized data of each physical memristor from their respective fits in Eqs. (S21) and (S22). Subsequently, we fit a normal distribution to these extracted values, as shown in Supplementary Fig. 11b. Due to the low number of points per cycle, a normal distribution does not exactly reproduce the histogram of the variations.

It should be noted that the fitted normal distribution tends to overestimate the noise impact: It better fits the tails of the distribution, which correspond to regions with higher update noise, while underestimating the central density, where the update noise is lower. Therefore, it contains fewer low-noise data points than actually measured.

When emulating the behaviour of memristors in software, we employ a constant update noise that is independent of the pulse index. This method is justified by examining the extracted noise from the potentiation and depression curves as a function of the pulse number, as depicted in Supplementary Fig. 11c. The data indicates little to no dependence on the pulse index.

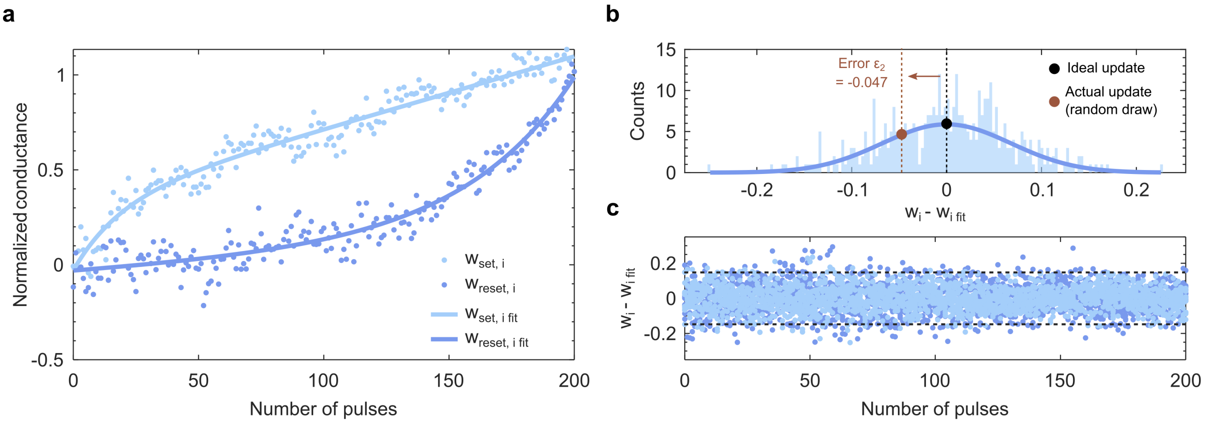

Supplementary Figure 11| Representative weight update and noise models used to emulate the behaviour of physical memristors in software. (a) Measured and fitted potentiation and depression curve for one cycle i. The four fitting parameters in Eqs. (S21) and (S22) take the values $\alpha_{set} = 0.33$, $\beta_{set} = 3.8 \times 10^{-3}$, $\gamma_{set} = 0.36$, $\zeta_{set} = 5.31 \times 10^{-2}$ for potentiation and $\alpha_{reset} = -3.96 \times 10^{-2}$, $\beta_{reset} = 8.38 \times 10^{-4}$, $\gamma_{reset} = 9.5 \times 10^{-3}$, $\zeta_{reset} = 2.25 \times 10^{-2}$ for depression. **(b)** Histogram showing the extracted deviations of the normalized potentiation and depression data from their respective means for the cycle shown in subfigure (a). A Gaussian distribution is assumed to reproduce this noise (dark blue). We draw a random value from this normal distribution to emulate the update noise in software. The presented example illustrates that the ideal weight update Δw (black dot) is offset by ϵ_2 (red dot), resulting in the actual update being $\Delta w + \epsilon_2$. **(c)** Extracted deviations of the measured potentiation (light blue points) and depression (dark blue points) data from their mean values for the 10 cycles that were measured as a function of the pulse index. The update noise exhibits little to no dependence on the pulse number. The black dotted lines serve as indicators that the majority of values fall within two standard deviations.

Cycle-to-cycle variability modeling in the memristor emulation

The weight updates on in-software-emulated memristors incorporate cycle-to-cycle variability as different cycles are chosen at each iteration. As mentioned in the main text, we measured 10 distinct potentiation and depression cycles for every device. Supplementary Fig. 12a shows the measured cycles along

with their fits for one representative memristor. The fits were made based on Eqs. (S21) and (S22). They serve as inputs to update the synaptic weights within the actor-critic temporal difference (TD) learning task and are utilized by the software whenever the artificial synapses should undergo weight updates.

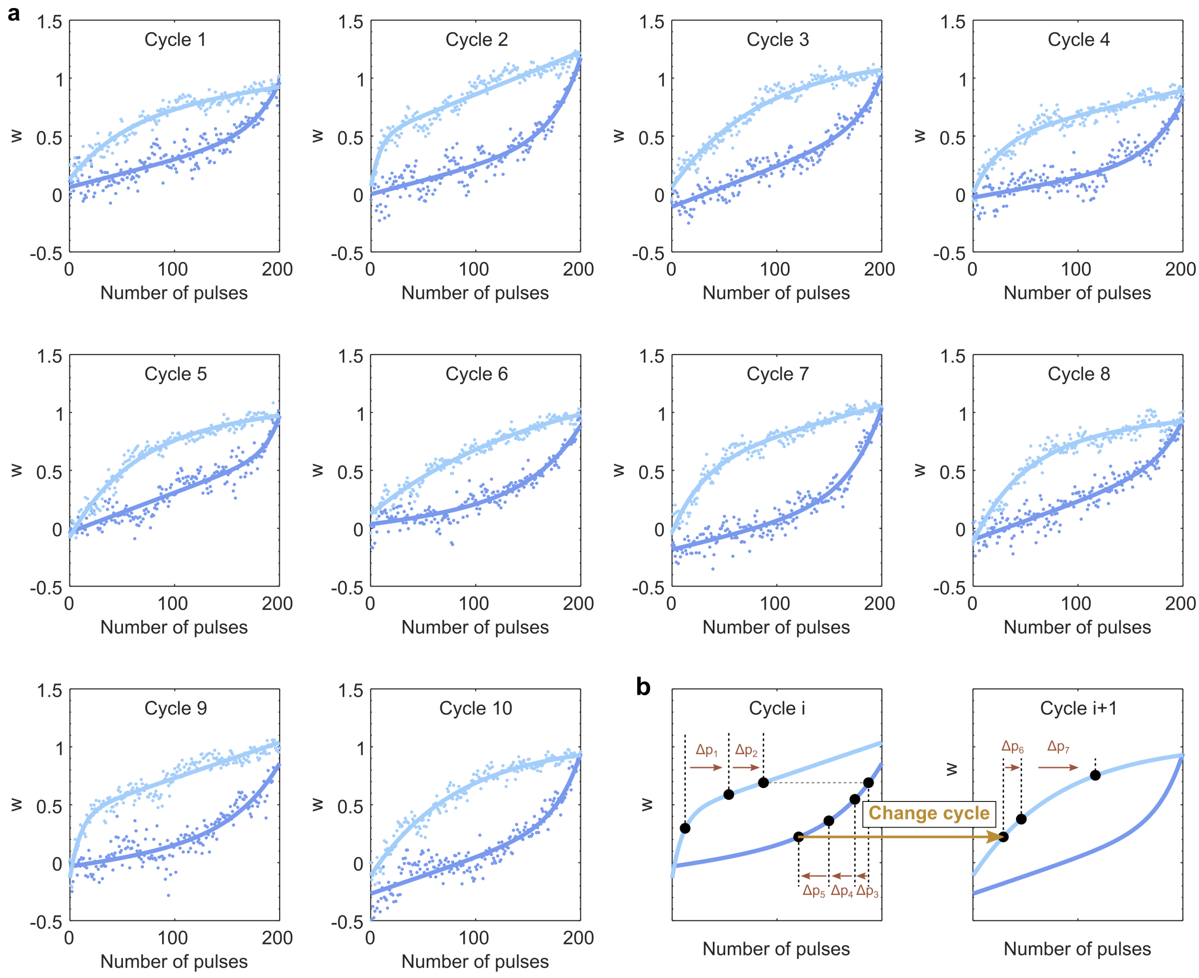

Supplementary Figure 12| Including cycle-to-cycle variability in the memristor emulation. (a) Measurement of 10 different potentiation and depression cycles for one device together with the corresponding fits according to Eqs. (S21) and (S22). The fitted curves are used to update the weights of memristors in software. **(b)** Illustration of the procedure to include cycle-to-cycle variability in software. Updates happen on the potentiation/depression curves of a certain cycle "i". A new cycle "i+1" is selected as soon as the sign of the update changes twice. Here, the first sign change happens from Δp_2 to Δp_3 (potentiation to depression). The second sign change occurs from Δp_5 to Δp_6 (depression to potentiation), which triggers a cycle change. The fitted curve of the new cycle is then employed for the subsequent weight updates, until two potentiation/depression transitions have again taken place.

We implemented cycle-to-cycle variability as described in Supplementary Fig. 12b. It is illustrated there how a cycle change from "i" to "i+1" happens during the weight update process. The updates are

performed on the same potentiation/depression cycle "i" until the sign of Δw has changed for the second time. When this occurs, the next weight update is performed with the fitted potentiation/depression curves of cycle "i+1". This serves as input for the weight updates during the next two Δw changes before moving to cycle "i+2".
In the presented example, the weight update process starts with two positive pulses (Δp_1, Δp_2) and is thus carried out on the potentiation curve. A sign change between Δp_2 and Δp_3 brings the weight update on the depression curve, where three (Δp_3, Δp_4, Δp_5) weight changes are executed. The sign change taking place between Δp_5 and Δp_6 requires to transition again to the potentiation curve. However, since this is the second time that the weight update changes its sign on cycle "i", the algorithm takes the potentiation curve from cycle "i+1" to apply the next updates (Δp_6, Δp_7).

The algorithm to change cycles every two potentiation/depression transitions is intended to mimic cycle-to-cycle variability in physical memristors. In our code, we start by selecting a random cycle "i" in between 1 and 10 (e.g., cycle 4). A variable $sign_{counter}$ keeps track of the sign changes. When it is equal to two, a new cycle is selected and $sign_{counter}$ is reset to zero. In doing so, the variable $count_{direction}$ defines the direction of selecting the next cycle. If $count_{direction} = up$, cycle "$i + 1$" is selected (e.g., cycle 5), whereas cycle "$i - 1$" (e.g., cycle 3) is chosen in case of $count_{direction} = down$. Initially, $count_{direction} = up$ and thus the cycles are increased in increments of 1. When cycle 10 is reached, the count direction is flipped (i.e., $count_{direction} = down$) and is counted down to cycle 1. There, the count direction is again flipped to $count_{direction} = up$. This procedure is described below in the pseudocode of Algorithm 1 for the case where the update is positive. In the case of a negative update, the only thing that changes is $update\_sign = -1$ (line 22), since the update sign is negative.

Algorithm 1: Cycle-to-cycle variability on in-software-emulated memristors

```
Data: sign_counter = 0, update_sign = None, cycle = None, count_direction = up,
      cycle_max = 10, Pulseupdate Δp
if update > 0 then
    if sign_counter == 0 and cycle == None then
        cycle ← Select random integer between 0 and cycle_max − 1 to initialize cycle;
        sign_counter ← 1;
    else if update_sign * Δp < 0 then
        if (sign_counter == 2) and (cycle > 0) and (cycle < cycle_max − 1) then
            sign_counter ← 1 // Reset counter
            if count_direction == up then
                cycle ← cycle + 1 // Switch to next cycle
            else if count_direction == down then
                cycle ← cycle − 1// Switch to next cycle
        else if sign_counter == 2 then
            // If cycle is at boundary, flip direction
            sign_counter ← 1 // Reset counter
            if count_direction == up then
                count_direction ← down;
                cycle ← cycle − 1// Switch to next cycle
            else if count_direction == down then
                count_direction ← up;
                cycle ← cycle + 1// Switch to next cycle
        else
            sign_counter ← sign_counter + 1 // Sign change, but counter not
                at 2
    update_sign = 1// Update sign is positive
```

Supplementary Note 4: Error correcting mechanism

Both the error terms ϵ_1 and ϵ_2 are directly included in the in-memory weight update calculation and thus contained in the weight update of the next iteration. As the weight updates associated with the TD error are calculated continuously, errors do not accumulate over time and can be trained away. In the case of one-hot encoding, two memristors with the weights w_{t+1} and w_t are involved in the calculation of the weight update that store the value estimates of the current and the next state. In other words, $V(s_{t+1}) = w_{t+1}$ and $V(s_t) = w_t$. Whenever there is a difference between actual and expected reward, a non-zero TD error occurs that leads to a (desired) weight update Δw_{des}. In the ideal case, the memristors would simply be updated according to:

$$w_{i,ideal} \leftarrow w_i + \Delta w_{des,i} \tag{S23}$$

where i denotes the memristor index (i.e., t or t+1). However, due to the error terms ϵ_1 and ϵ_2 the actual update is given by:

$$w_{i,actual}(\epsilon_{1,i}, \epsilon_{2,i}) \leftarrow w_i + \Delta w_{des,i} + \epsilon_{1,i} + \epsilon_{2,i} \tag{S24}$$

where $\epsilon_{1,i}$ is the error term that arises from performing linear updates on non-linear update curves and $\epsilon_{2,i}$ corresponds to the update noise. $\epsilon_{1,i}$ and $\epsilon_{2,i}$ are included into weight update calculation of the next iteration as they are contained in the conductance values of $w_{i,actual}$:

$$\begin{aligned}\Delta w_{des}(\epsilon_{1,i}; \epsilon_{2,i}) &= \alpha\left(U_1 \times r(s_t) + U_2 \times w_{t+1,actual}(\epsilon_{1,t+1}; \epsilon_{2,t+1}) + U_3 \times w_{t,actual}(\epsilon_{1,t}; \epsilon_{2,t})\right) \\ &= \underbrace{\alpha \times U_1 \times r(s_t) + \alpha \times U_2 \times w_{t+1} + \alpha \times U_3 \times w_t}_{\Delta w_{ideal}} \\ &\quad + \underbrace{\alpha \times U_2 \times (\epsilon_{1,t+1} + \epsilon_{2,t+1}) + \alpha \times U_3 \times (\epsilon_{1,t} + \epsilon_{2,t})}_{\Delta w_{corr}}\end{aligned} \tag{S25}$$

where U_i are the applied voltages in the hardware error calculation (Eqs. (S11)-(S13)). The second equation highlights the component of the weight update Δw_{des} coming directly from the error in $\epsilon_{1,i}$ and $\epsilon_{2,i}$. As the TD error is calculated continuously, any error arising due to $\epsilon_{1,i}$ and $\epsilon_{2,i}$ is thus repeatably trained away directly in hardware.

If no error correction mechanism was present, the weight update Δw_{des} would depend on the ideal conductance values $w_{i,ideal}$ only and it would not consider the error terms ϵ_1 and ϵ_2.

Supplementary Note 5: Grid search heatmaps for memristors with ideal (linear) and measurement-driven (non-linear, noise, variability) weight updates

To identify the optimal hyperparameters for the hardware runs, we conducted grid searches with the T-maze navigation task of Fig. 4 of the main text where the key learning parameters were varied and memristor emulations were used: α (learning rate) and T (temperature factor of the softmax policy). The software runs we performed encompass the following scenarios:

- **Ideal case:** Ideal updates, linear dependence of Δw on Δp, no update noise
- **Emulated case without update noise:** Updates following the fitted curves extracted from random device cycles, inclusion of cycle-to-cycle variability, no update noise
- **Emulated case with update noise:** Updates following the fitted curves extracted from random device cycles, inclusion of cycle-to-cycle variability and update noise

In all these cases, we assumed a linear relation between Δp and Δw for calculating the number of pulses corresponding to the weight update. These ”ideal” pulse updates can be executed on ideal synaptic weights (ideal update), or on one of ten distinct weight model curves per device (memristor model with cycle-to-cycle variability).
Additionally, we consider two different variants of the memristor emulations. One case where the model does not include update noise and one case where it is included. The update noise in the latter case is different for each cycle, as explained in Supplementary Note 3.

Subsequently, we compare the grid searches for all three cases outlined above and evaluate the results by means of two different metrics for the actor and critic networks. This dual-metric approach is essential because the softmax temperature hyperparameter directly regulates the balance between exploration and exploitation [13] and thus impacts the learning strategy: In the case of a low softmax temperature, the actor primarily focuses on exploiting the knowledge and policies it has acquired, resulting in low exploration. The latter implies that the values of unvisited states are not learned. On the other hand, a high softmax temperature leads to increased exploration, motivating the actor to explore different strategies and potentially discover more effective approaches. However, this comes at a cost of slower convergence, i.e., more step/episodes are needed.

Our two metrics should thus reflect both those cases: The first metric should assess the effectiveness of exploitation, evaluating how well the actor leverages its existing knowledge, whereas the second metric should focus on exploration. Straightforward, yet effective metrics consist of evaluating the learned weights for both the actor and the critic by comparing them to their optimal values. By calculating the Root Mean Square Error (RMSE) between the learned values and their corresponding optimal values, we can quantify the average difference between these two quantities, providing a robust measure of the

learning performance. We define the actor and critic RMSE for the T-maze navigation task as follows:

$$RMSE_{actor} = \sqrt{\frac{1}{7}\sum_{i=1}^{n=7}(\theta_i - \hat{\theta}_i)^2} \tag{S26}$$

$$RMSE_{critic} = \sqrt{\frac{1}{9}\sum_{i=1}^{n=9}(w_i - \hat{w}_i)^2} \tag{S27}$$

where θ_i, w_i are the learned actor and critic weights, respectively, and $\hat{\theta}_i$, $\hat{w}_i$ correspond to their optimal counterparts. Note that θ_i is the net action per state i resulting from the difference of the two learned actions weights per state: $\theta_i = abs\,(\theta_{i,0} - \theta_{i,1})$. There are nine states in the environment of the T-maze navigation task (Supplementary Fig. 13a). For the RMSE of the actor, we only take the weights associated with states 0 to 6 into the calculation, as we want to evaluate how well the actor exploits the optimal trajectory that goes from the start (state 0) directly to the reward (state 6). The weights of states 7 and 8 are not included in the calculation as these states are only learned in case the softmax temperature is non-zero. Hence, they do not contribute to the stereotypical trajectory. By including them, we would penalize the situations where the actor follows the optimal trajectory.

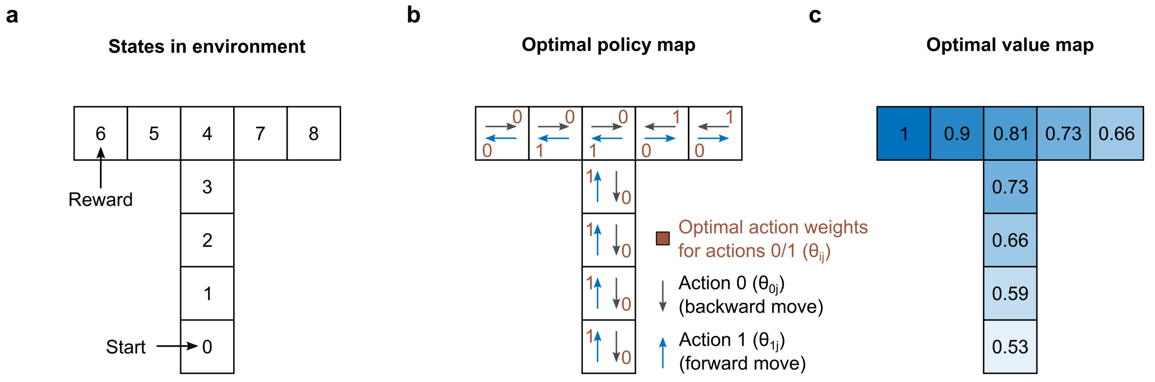

Supplementary Figure 13| States of the T-maze environment together with the optimal policy (θ) and value map (w). (a) States in the T-maze navigation task of Fig. 4 in the main manuscript. The agent starts at state 0 and attempts to locate the reward at state 6. **(b)** Optimal policy map with each state having two actor weights (θ_{0j} and θ_{1j}) associated. For each state, the actor weight is equal to 1 for the optimal action (blue arrows), while the weight corresponding to the "wrong" action is equal to zero (grey arrows). At the reward, both weights are zero since no actions are taken there. **(c)** Optimal value map for all nine states. The optimal value of the reward (state 6) is equal to one. Moving back in the environment, this value is discounted by the discount factor $\gamma = 0.9$ at every state. In other words, the value of the reward's nearest neighbour state is $1 \times \gamma = 0.9$, the value of the reward's second nearest neighbour state is $1 \times (\gamma = 0.9)^2$, the value of the reward's third nearest neighbour state is $1 \times (\gamma = 0.9)^3$, and so on.

For the RMSE of the critic, we take the weights associated with all nine states (i.e. state 0 to 8) into the calculation, as we want to evaluate how well the critic explores the environment. In this case, it

makes sense to include the weights of states 7 and 8 in the calculation as they contribute to the environment.

We can define the state vector as $[0, 1, 2, 3, 4, 5, 6, 7, 8]$. For the states 0 to 5 the optimal action is a forward move (action 1), while it is backward (action 0) for the states 7 and 8. For state 4 the agent can move to the left or right. Here, we define a movement to the left (in the direction of the reward) as a forward movement (action 1) and the one to the right (away from the reward) as a backward movement (action 0). Based on this definition, the optimal actor weights $\hat{\theta}_i$, illustrated in Supplementary Fig. 13b, are then given by $\hat{\theta} = [[0, 1], [0, 1], [0, 1], [0, 1], [0, 1], [0, 1], [0, 0], [1, 0], [1, 0]]^T$. On the other hand, the optimal critic weights $\hat{w}_i$, shown in Supplementary Fig. 13c, are equal to
$\hat{\mathbf{w}} = [\gamma^6, \gamma^5, \gamma^4, \gamma^3, \gamma^2, \gamma^1, \gamma^0, \gamma^3, \gamma^4] = [0.53, 0.59, 0.66, 0.73, 0.81, 0.9, 1, 0.73, 0.66]$ with $\gamma = 0.9$. Supplementary Figure 14 shows the grid searches of the $RMSE_{actor}$ and $RMSE_{critic}$ metrics as a function of the hyperparameters α and T for all aforementioned cases. The ideal case in Supplementary Fig. 14a nicely illustrates the exploitation characteristic of the actor and the exploration behaviour of the critic, emphasizing the trade-off between exploitation and exploration. The RMSE of the actor is the lowest when the temperature factor is the smallest. This makes sense because the actor RMSE quantifies how well the optimal policy is exploited. For cases with higher temperatures, more random actions are taken which results in increased RMSE values. On the other hand, the learning rate controls how fast the weights are updated based on new information. For the actor, a higher learning rate results in a faster convergence to the optimal policy and thus lower RMSE values.

In contrast to the actor, the RMSE of the critic is the lowest in case of highest temperature factor. This can be attributed to the fact that the critic RMSE quantifies the exploration of the environment. Simulation runs with higher temperatures lead to increased exploration. Consequently, the critic weights associated with states that do not lie not along the optimal trajectory (i.e., the direct path from state 0 to state 6) receive more training compared to cases with lower temperatures. Moreover, the learning rate is optimal when it is kept moderate to values between 0.2 and 0.4. A high learning rate generally implies that new information is weighted more heavily. When combined with a high temperature factor, this can cause the reinforcement of ”wrong” random actions, resulting in an overall increase in the critic’s RMSE compared to the case with lower learning rates.

Supplementary Fig. 14b depicts the RMSE of the actor and critic in the case of 27 in-software-emulated memristors with cycle-to-cycle variability. The actor RMSE exhibits the same trend for the different combinations of learning rates and temperatures, but the RMSE values are generally higher than those of the ideal case. This increase in RMSE is primarily due to the introduction of the error term, ϵ_1, resulting from the replacement of ideal synaptic weights updates by non-ideal ones. As discussed in the main text, this error arises from the difference between the ideal linear update and the non-linear weight update.

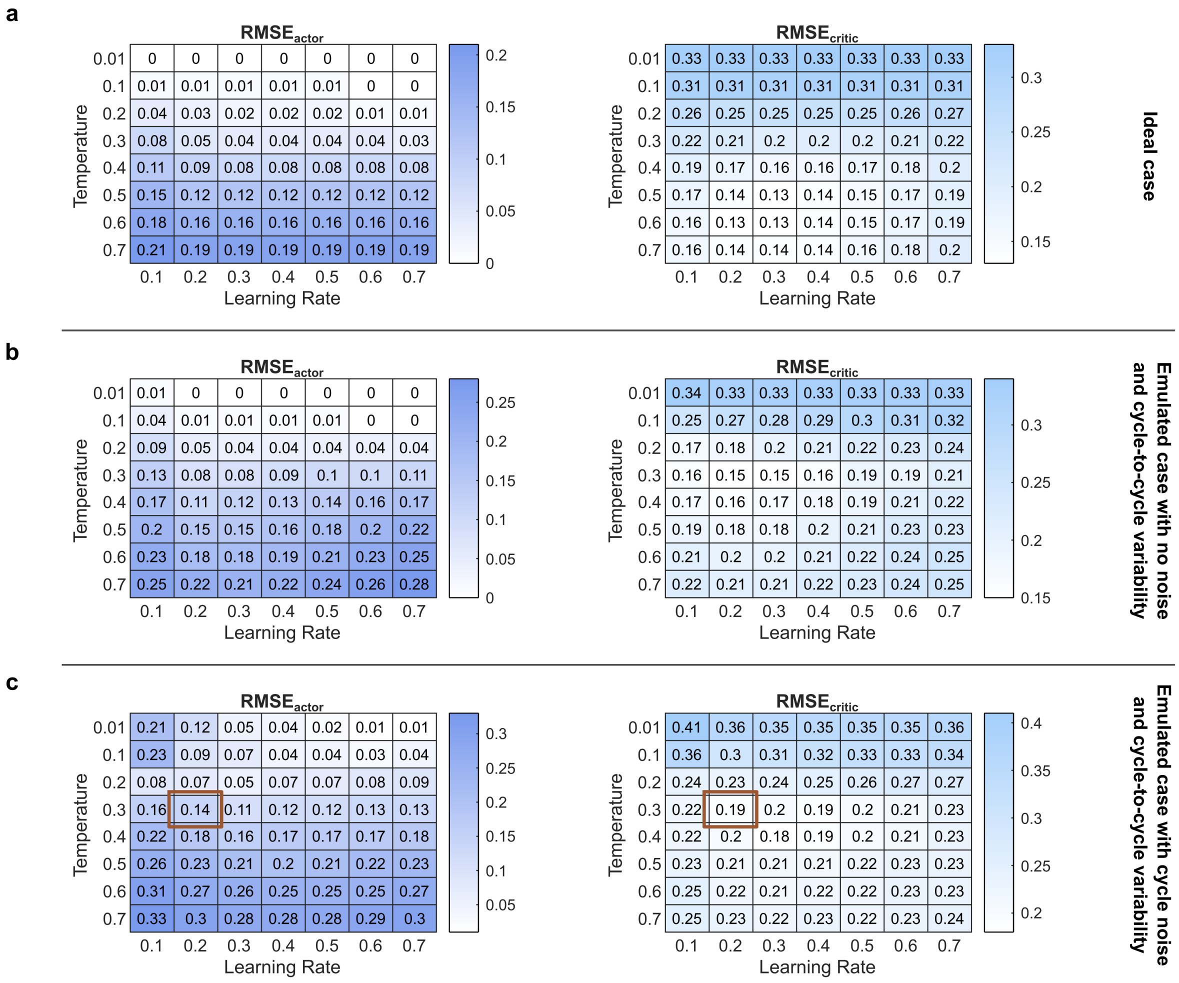

Supplementary Figure 14| Grid searches of the $\text{RMSE}_{\text{actor}}$ and $\text{RMSE}_{\text{critic}}$ metrics as a function of the learning rate (α) and temperature factor (T) hyperparameters. Each value is the average of 100 distinct simulation runs with 200 episodes. The values for $\text{RMSE}_{\text{actor}}$ and $\text{RMSE}_{\text{critic}}$ are extracted using Eqs. (S26) and (S27) at the end of the last episode. **(a)** Ideal case: perfectly linear weight updates, no noise, no cycle-to-cycle variability. **(b)** Non-ideal weight updates using the fitted potentiation/depression curves, including cycle-to-cycle variability, but no update noise. **(c)** Same as (b), but with update noise. The red boxes indicate the optimal hyperparameter combinations, i.e., those leading to the smallest $\text{RMSE}_{\text{actor}}$ and $\text{RMSE}_{\text{critic}}$ that are used in Fig. 4 of the main text.

Similarly, the error term ϵ_1 affects the critic RMSE values as well, causing them to be higher than in the ideal case. This indicates greater deviation of the learned weights from their optimal values. Moreover, it can be observed that the optimal RMSE critic value is obtained at lower temperature values, with the optimum at $T = 0.3$. This shift is attributed to the combination of increased exploration (high temperature values) and the influence of ϵ_1, which can cause the trained critic weights to deviate from their correct values, resulting in a higher RMSE. Additionally, the inclusion of cycle-to-cycle variability could also result in suboptimal actor and critic weight values, contributing to increased RMSE values. Nonetheless, the introduction of non-linear updates and cycle-to-cycle variability into the software does not significantly affect the simulations to converge, which remains similar to the ideal case.

Finally, Supplementary Fig. 14c illustrates the RMSE of the actor and critic networks, including 27 different in-software-emulated memristors with non-linear weight updates, cycle-to-cycle variability, and update noise. As discussed in the main text, the update noise obeys a normal distributions with parameters extracted by fitting experimental data. An error term ϵ_2 is introduced in the simulations as the weight update processes are disturbed by random draws from these normal distributions. The resulting error causes both actor and critic weights to deviate from their learned values, which impacts the whole learning process. Consequently, we observe increased RMSE values for both actor and critic compared to the case without cycle noise. Despite the added complexity, the approach still works, all simulations converging in a similar manner as in the case without cycle noise.

For our hardware runs in Fig. 4 of the main text, we use a learning rate of 0.2 and a temperature factor of 0.3 (red box in Supplementary Fig. 14c). The latter should be large enough to ensure enough exploration. Optimal RMSE values, as seen in the right plot of Supplementary Fig. 14c, are achieved at temperature factors of 0.3 or 0.4. Since actors tend to prefer lower temperatures for exploitation, we select the smaller value (i.e., 0.3) for T. Regarding the learning rate, values between 0.2 and 0.5 yield reasonable RMSE values for both the actor and critic networks. To minimize the impact of the linear pulse updates in our in-software-emulated memristors (associated with the error term ϵ_1), we opt for a learning rate of 0.2. As mentioned above, lower learning rates prioritize existing knowledge over new information and thus reduce the sensitivity to error terms.

Supplementary Note 6: T-maze navigation task using hardware actor weights

Besides the nine critic weights, we also conducted online training on all actor weights, utilizing pairs of physical memristors. Supplementary Fig. 15a shows the action weights as a function of the episode number. To implement all actor weights in hardware, nine distinct configurations were tested. Both actions associated to a specific state were implemented by two distinct physical memristors, while the behaviour of the other actor and all critic weights was emulated in software incorporating cycle-to-cycle variability. In doing so, each of the measured actor weights was represented by a different physical memristor (see also Extended Data Fig. 3). A comparison between the measured curves and the software runs indicates an overall good agreement. Similar to the critic weights (Fig. 4 of the main text), some deviations are observed that can be related to fluctuations related to the non-ideal potentiation/depression curves (error term ϵ_1) and update noise (error term ϵ_2).

Supplementary Figure 15b displays the final trained action values extracted from the measured devices. They align well with values extracted from software runs that use ideal synaptic weights (continuous, perfectly linear, and no noise). Most importantly, at each measured state, the correct action is learned while the incorrect one remains zero.

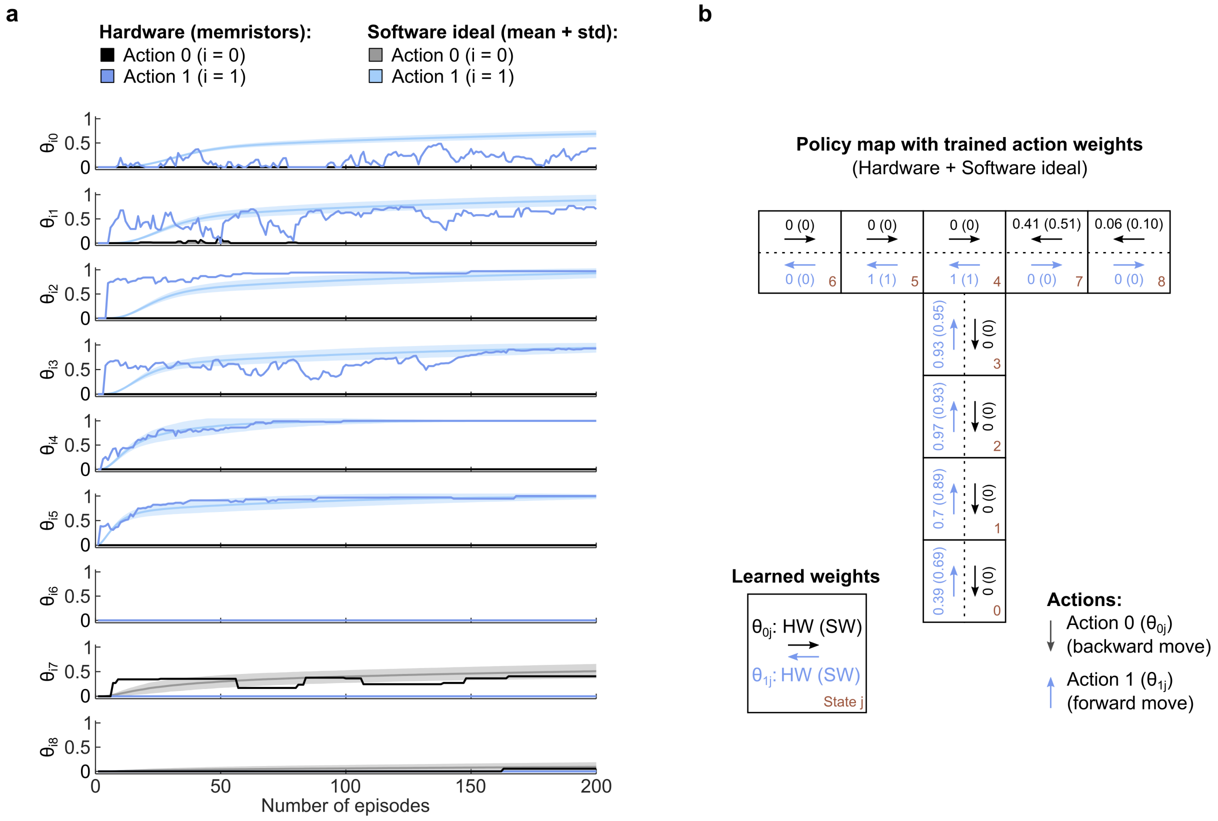

Supplementary Figure 15| Trained hardware actor weights of the T-maze navigation task. (a) Measured actor weights over 200 episodes compared to the ideal software case. For each run, the two possible actions of a particular state were implemented on two physical memristors and updated in hardware via online training. The software runs are the average of 1000 runs with error bars indicating two standard deviations. Note that action 0 for states 0 to 6 and action 1 for states 7 and 8 remain zero and are the same for both the hardware and software case. **(b)** Policy map with trained actor weights for both the hardware and the ideal software case (continuous, linear, and no noise). The actor weights of all states were trained on hardware memristors and the final values after 200 episodes of the runs in (a) were extracted. Note that there are two actor weights for each state, one per action: moving forward/backward for all states, except for state 4 (moving left/right). The corresponding hardware and software (between parentheses) values are provided for each measured action.

Supplementary Note 7: TD learning framework on crossbar arrays for higher dimensional input activations

In the T-maze navigation task presented in Fig. 4 of the main text, our framework was tested in the simplified case of one-hot encoding, where only one entry of the input vector is non-zero (see also Supplementary Note 1). In the Morris water maze navigation task (Fig. 5 of the main text), learning takes place in continuous state space and the assumption of one-hot encoding does not hold anymore. There, multiple elements of the input activation vector $\mathbf{x_t}$ are non-zero and it holds:

$$\sum_{s=1}^{N_{grid\,points}} x_{t,s} = 1 \tag{S28}$$

where the $x_{t,s}$'s are the elements of the input activation vector, s is a particular state of the environment, and $N_{grid\,points}$ is the total amount of grid points in the environment. The activation $\mathbf{x_t}$ is given by several overlapping place cells described as Gaussian Radial Basis Functions (RBFs). The presented formulas in Section 2.1 (i.e., Eqs. (1) to (3)) of the main text are valid for the general case of continuous state spaces where $\mathbf{x_t}$ is a vector of size $N_{grid\,points}$. However, continuous space representations have direct implications on the TD learning process using memristors:

- The action choice (activity) $\mathbf{h}$, which has size m (with m being the number of possible actions), is given by the matrix-vector multiplication: $\mathbf{h} = \Theta \cdot \mathbf{x_t}$, where Θ is the $N_{grid\,points} \times m$ action matrix and $\mathbf{x_t}$ is the input activation.
- The third factor (or TD error) 3rd (δ_t) required for the in-memory weight update calculations of $\Delta\mathbf{w}$ and $\Delta\boldsymbol{\theta}$ is obtained by performing the vector-vector multiplication of $(\gamma \times \mathbf{x_{t+1}} - \mathbf{x_t}) \cdot \mathbf{w}$, where γ is the discount factor, $\mathbf{x_{t+1}}$ ($\mathbf{x_t}$) the input activation of the state at time $t+1$ (t), and $\mathbf{w}$ the vector containing all critic weights.

Weight update calculation and action computations in a crossbar array

Both the action choice and the weight updates associated with the TD error can be computed very efficiently using crossbar arrays, which is described and illustrated in the following. The crossbar array has $m+1$ columns (m actions and 1 critic) and $n = N_{grid\,points}$ rows (see Supplementary Fig. 16). The critic weights are assigned to the first column, whereas the action weights are mapped to the other columns (i.e., columns 2 to $m+1$). The ideal crossbar array architecture for such an implementation would be a 1T1R structure to avoid the issue of sneak path currents. However, for the sake of simplicity, we omitted the transistors in Supplementary Fig. 16. The details of a 1T1R implementation as well as the IR drop in this architecture are discussed below.

The agent samples its actions based on the action probabilities $\pi(i|s_t)$ at a distinct state s_t. $\pi(i|s_t)$ depends on the activity $\mathbf{h}$, which can be calculated very efficiently in a single step using crossbar arrays (see Supplementary Fig. 16a), as it is the result of a matrix-vector multiplication.

The agent then samples an action a_t from $\pi(i|s_t)$, makes a step in the environment, and observes the reward $r(s_t)$ at that state. The difference between the actual and expected reward leads to a non-zero TD error 3rd (according to Eq. (2) of the main text) that updates the actor and critic weights θ_{ij} and w_j (according to Eq. (1) of the main text). More precisely, the weight updates for both the actor ($\Delta\boldsymbol{\theta}$) and critic weights ($\Delta\mathbf{w}$) are proportional to the product of the learning rate α and TD error 3rd. This product can be computed in a single step by performing a vector-vector multiplication using the column with the critic weights (see Supplementary Fig. 16b). The effective actor and critic update vectors are then obtained by multiplying the result ($\alpha \times 3^{rd}$) with the corresponding Hebbian term.

IR drop in a 1T1R crossbar implementation

Next, we discuss a possible hardware implementation of the Morris water maze task of the main text using an 1T1R crossbar array and calculate the maximum IR drop. For this implementation, a crossbar array with dimension 121×9 (11×11 place cells, 8 actions + 1 value per place cell) is needed. A 1T1R configuration with transistor selector lines along the columns is chosen, depicted in Supplementary Fig. 17. The influence of access transistors (1T) was not explicitly accounted for in this work. The on-state resistance of the transistor is typically below 1 kΩ (e.g., 0.8 kΩ in [14]), effectively acting as a series resistance and dynamic voltage divider. It plays a role especially at the onset of and during the set process itself, which can improve the overall switching reliability, as demonstrated in [14] and [15]. To calculate the IR drop we can consider the following parameters for the crossbar array:

- Tungsten wires (same material as top electrodes) of width w_{wire} = 600 nm (active area of devices used in this work is 600 nm × 600 nm) and thickness t_{wire} = 150 nm (thickness of top electrode)
- Device pitch of $pitch$=1.2 µm (assumed to be twice the width of a device)
- The number of columns is N_{cols}=9

Based on this data, we can investigate the worst-case IR drop in the resulting crossbar array. This occurs in a parallel readout, where all bit lines (BL) are driven by $V_{read} = 0.2$ V, all transistors are active, and all devices are in their low resistance state (LRS) with $G_{i,LRS}$=50 µS. Note that we do not expect a substantial voltage drop across the access transistor, as its on-resistance (i.e., below 1 kΩ) is significantly lower than the LRS of the memristors (15-20 kΩ at a read voltage of 0.2 V). Therefore, no increase in the operating voltage is expected to be necessary. Since all transistors are selected, we have the same situation as in a passive crossbar array. The largest IR drop occurs for a readout of the last device of each row. As all rows are the same, we can consider row 1 for the calculation of the IR drop, indicated in blue in Supplementary Fig. 17. In this scenario the following resistances are involved:

$$R_{wire} = \rho_w \frac{l_{wire}}{A_{wire}} = 6.7\Omega \tag{S29}$$

$$R_{VCM,tot} = \frac{1}{\sum_{i=1}^{N_{cols}} G_{i,LRS}} = 2222.2\Omega \tag{S30}$$

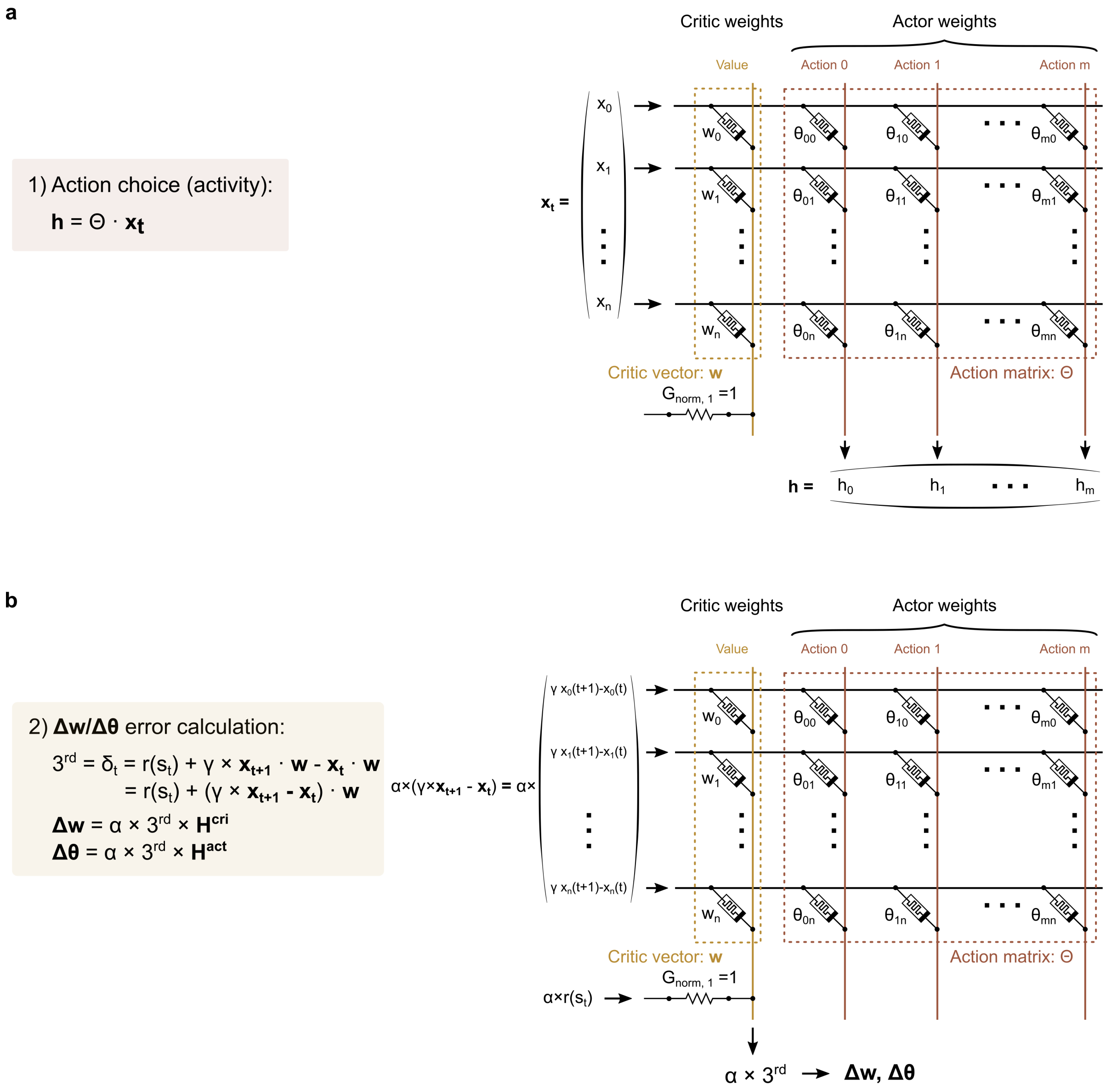

Supplementary Figure 16| TD learning framework on crossbar arrays (a) Action probabilites (activity) calculation on a crossbar array. The activity vector **h** is the result of a matrix-vector multiplication when applying the input vector $\mathbf{x_t}$ at the rows and reading out the current at the columns of the action matrix Θ. One single action i is mapped to a column of the crossbar array. **(b)** weight update calculation on a crossbar array. This operation involves computing the term $\alpha \times 3^{rd}$, which results from a vector-vector multiplication where the input vector $\alpha \times (\gamma \times \mathbf{x_{t+1}} - \mathbf{x_t})$ is applied to the rows and the current at the column of the critic vector **w** is read out. In the presence of a rewarding event, an additional term $\alpha \times r(s_t)$ is added to the result using a fixed resistor, similarly to the one-hot case depicted in Fig. 3a of the main text. The weight updates for both the actor ($\Delta\boldsymbol{\theta}$) and critic weights ($\Delta\mathbf{w}$) are then determined by the multiplication of their corresponding Hebbian terms. These updates are not single elements, but vectors, which could allow for the simultaneous updating of all rows.

where $l_{wire} = N_{cols} \times pitch$, $A_{wire} = t_{wire} \times w_{wire}$ is the rectangular cross section, and ρ_w=5.6× 10^{-9} Ω m is the resistivity of tungsten. This results in the following IR drop:

$$IR_{drop} = \frac{R_{wire}}{R_{wire} + R_{VCM,tot}} = 0.3\% \tag{S31}$$

Clearly, the IR drop is very small. Therefore, we do not expect a significant voltage drop for the crossbar array size considered here such that the global TD error can be broadcast to each synaptic element. Moreover, even in larger arrays, as in the case of a larger maze or an increased number of possible actions, the convergence of the algorithm would not be hindered since it is very robust to the different sources of noise that exist, as shown in Extended Data Fig. 4.

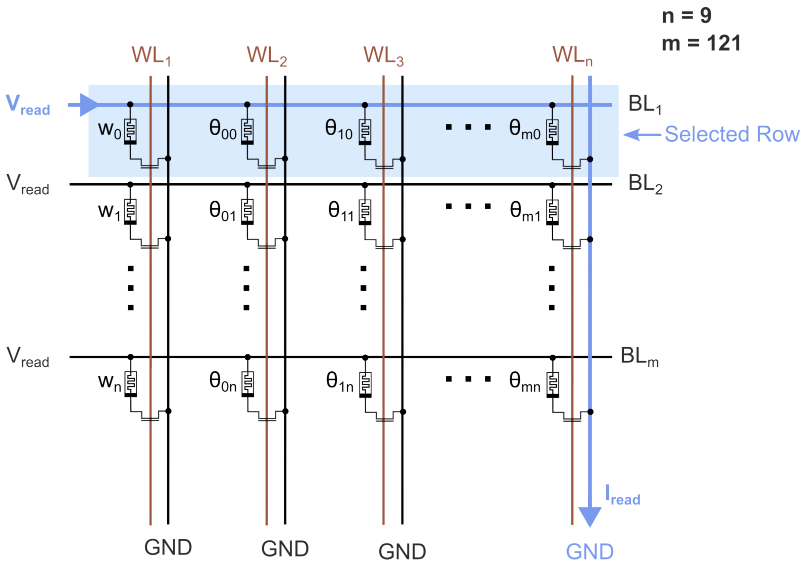

Supplementary Figure 17| IR drop in a 1T1R crossbar array implementation of the actor-critic network in the Morris water maze task The 1T1R crossbar array has the dimension 121 × 9 corresponding to 11 × 11 place cells and 8 actions + 1 value per place cell, with transistor word lines (WL_i) along the columns. In the worst-case scenario, a parallel readout of all rows is performed and all devices are in the low resistance state $G_{i,LRS}$=50 µS. In this scenario all transistors are active which leads to the same situation as in a passive crossbar array. The largest IR drop occurs at the last device of each row which leads to the largest wire resistance R_{wire}. As all rows are the same, we can consider row 1 indicated in blue. Since all transistors are active, all the memristors in the selected row have a parallel connection to ground which leads to the resistance $R_{VCM,tot}$.

Supplementary Note 8: Energy consumption during the in-memory weight training of the T-maze task

We calculated the worst-case energy consumption during in-memory training of the T-maze task on hardware memristors. The energy consumed consists of two parts: weight updates and the in-memory weight update calculation. To estimate the energy consumption of the weight updates, we extracted all updates on the 27 hardware memristor that were performed during the T-maze task from the measurements. For simplicity and to estimate the upper limit of the energy consumption, we then assumed that all updates occur when the devices are in their highest conductance state, $G_{max} = 50$ µS. This results in the following formulas for the energy consumption of all weight updates:

$$E_{set} = n_{set} \times U_{set}^2 \times t_{set} \times G_{max} \tag{S32}$$

$$E_{reset} = n_{reset} \times U_{reset}^2 \times t_{reset} \times G_{max} \tag{S33}$$

$$E_{updates,tot} = E_{set} + E_{reset} \tag{S34}$$

where $n_{set} = 5642$ and $n_{reset} = 2712$ are the total number of potentiation and depression pulses, respectively, that were applied to the 27 memristors, $U_{set} = 2.5$ V and $U_{reset} = -2.7$ V are the programming voltages, and $t_{set} = 1.5$ µs and $t_{reset} = 10$ µs are the corresponding pulse widths. This results in an energy consumption of 0.47 nJ for each set pulse, 3.65 nJ for each reset pulse, or in total 12.53 µJ for all 2264 weight updates. However, in principle, the memristor technology in this work can be programmed with 60 ns on a high-speed measurement setup [16]. This would allow for a drastic reduction of the energy consumption to just 0.17 µJ.

Similarly, we calculated the energy consumption of the hardware calculation of Δw. First, we extracted the total number of these operations performed during the T-maze task from the measurement data. We then assumed a scenario where each update is $\Delta w = 0.2$, which is the maximum value that it can take in this work with the learning parameters $\alpha = 0.2$ and $\gamma = 0.9$. This can be best understood by considering the expression for Δw:

$$\Delta w = \alpha \left((s_t) + \gamma \times V(s_{t+1}) - V(s_t) \right) \tag{S35}$$

where the worst-case scenario corresponds to the case $r(s_t) = 1$, $V(s_{t+1}) = V(s_t) = 0$ (note that $V(s_{t+1})$ is always zero at the reward, so the maximum value of bracket is 1). In the hardware calculation of Δw this equation becomes Eq. (S10) in the Supplementary Note 2:

$$\Delta w = U_1 \times G_{fixed} + U_2 \times G_{t+1} + U_3 \times G_t \tag{S36}$$

where G_{fixed} corresponds to the 10 kΩ resistor, and G_{t+1} and G_t are the conductance values of state $V(s_{t+1})$ and $V(s_t)$. The applied voltages are given by Eqs. (S11)-(S13) in Supplementary Note 2. As an amplifier with a $gain$ =10^5 is used, the voltages are scaled by this factor, which prevents any change of the memristor's resistance state:

$$U_1 = \frac{1}{G_{fixed} \times gain} \times \left(\alpha \times r(s_t) - \frac{\alpha(1-\gamma)G_{min}}{G_{max} - G_{min}} \right) \tag{S37}$$

$$U_2 = \frac{1}{gain} \times \frac{\alpha \times \gamma}{G_{max} - G_{min}} \tag{S38}$$

$$U_3 = \frac{1}{gain} \times \frac{-\alpha}{G_{max} - G_{min}} \tag{S39}$$

In the described scenario, we assume $r(s_t) = 1$, $G_{t+1} = G_t = G_{max}$ and $G_{min} = 30$ µS. Using these formulas, we then calculated the total energy consumption:

$$E_{\Delta w,tot} = n \times t_{read} \times \left(U_1^2 \times G_{fixed} + U_2^2 \times G_{max} + U_3^2 \times G_{max}\right)$$

where n is the total number of Δw calculations and t_{read}=20 ms is the voltage pulse duration of the Δw calculation. This results in an energy consumption of 17.26 nJ per Δw calculation and a total of 15.70 µJ for the entire T-maze task. Again, the energy consumption could be drastically reduced by decreasing the pulse widths. Pulses of 60 ns would result in 51.77 fJ per calculation, and a total energy consumption of 47.11 pJ. Overall, we end up with the following energy consumption for the T-maze task (depending on the used pulse durations):

- Current implementation: $E_{tot} = 12.53\mu J + 15.70\mu J = 28.23\mu J$
- Improved pulse widths (60 ns): $E_{tot} = 0.17\mu J + 47.11pJ = 0.17\mu J$

References

[1] Yuji Takahashi, Geoffrey Schoenbaum, and Yael Niv. "Silencing the critics: understanding the effects of cocaine sensitization on dorsolateral and ventral striatum in the context of an actor/critic model". In: *Frontiers in neuroscience* 2 (2008), p. 282.

[2] H-S Philip Wong et al. "Metal–oxide RRAM". In: *Proceedings of the IEEE* 100.6 (2012), pp. 1951–1970.

[3] Bernd Illing et al. "Local plasticity rules can learn deep representations using self-supervised contrastive predictions". In: *Advances in neural information processing systems* 34 (2021), pp. 30365–30379.

[4] Md Alam, Chris Yakopcic, and Tarek M Taha. "On-Chip Optimization and Deep Reinforcement Learning in Memristor Based Computing". In: *Proceedings of the 18th ACM International Symposium on Nanoscale Architectures*. 2023, pp. 1–7.

[5] S Bianchi et al. "A self-adaptive hardware with resistive switching synapses for experience-based neurocomputing". In: *Nature Communications* 14.1 (2023), p. 1565.

[6] Yuehua Dai et al. "Intrinsic decay property of Ti/TiOx/Pt memristor for reinforcement learning". In: *Advanced Intelligent Systems* 5.7 (2023), p. 2200455.

[7] Thomas Dalgaty et al. "In situ learning using intrinsic memristor variability via Markov chain Monte Carlo sampling". In: *Nature Electronics* 4.2 (2021), pp. 151–161.

[8] Yudeng Lin et al. "Uncertainty quantification via a memristor Bayesian deep neural network for risk-sensitive reinforcement learning". In: *Nature Machine Intelligence* 5.7 (2023), pp. 714–723.

[9] Yingming Lu et al. "In-memory realization of eligibility traces based on conductance drift of phase change memory for energy-efficient reinforcement learning". In: *Advanced Materials* 34.6 (2022), p. 2107811.

[10] Zhongrui Wang et al. "Reinforcement learning with analogue memristor arrays". In: *Nature electronics* 2.3 (2019), pp. 115–124.

[11] Pai-Yu Chen, Xiaochen Peng, and Shimeng Yu. "NeuroSim: A circuit-level macro model for benchmarking neuro-inspired architectures in online learning". In: *IEEE Transactions on Computer-Aided Design of Integrated Circuits and Systems* 37.12 (2018), pp. 3067–3080.

[12] Jacopo Frascaroli et al. "Evidence of soft bound behaviour in analogue memristive devices for neuromorphic computing". In: *Scientific reports* 8.1 (2018), p. 7178.

[13] Richard S Sutton and Andrew G Barto. *Reinforcement learning: An introduction*. MIT press, 2018.

[14] Donato Francesco Falcone et al. "All-in-One Analog AI Hardware: On-Chip Training and Inference with Conductive-Metal-Oxide/HfOx ReRAM Devices". In: *Advanced Functional Materials* (), p. 2504688.

[15] Tommaso Stecconi et al. “Filamentary TaOx/HfO2 ReRAM Devices for Neural Networks Training with Analog In-Memory Computing”. In: *Advanced electronic materials* 8.10 (2022), p. 2200448.

[16] Davide GF Lombardo et al. “Read Noise Analysis in Analog Conductive-Metal-Oxide/HfO x ReRAM Devices”. In: *2024 Device Research Conference (DRC)*. IEEE. 2024, pp. 1–2.
